# Lesion location implemented magnetic resonance imaging radiomics for predicting *IDH* and *TERT* promoter mutations in grade II/III gliomas

**DOI:** 10.1038/s41598-018-30273-4

**Published:** 2018-08-06

**Authors:** Hideyuki Arita, Manabu Kinoshita, Atsushi Kawaguchi, Masamichi Takahashi, Yoshitaka Narita, Yuzo Terakawa, Naohiro Tsuyuguchi, Yoshiko Okita, Masahiro Nonaka, Shusuke Moriuchi, Masatoshi Takagaki, Yasunori Fujimoto, Junya Fukai, Shuichi Izumoto, Kenichi Ishibashi, Yoshikazu Nakajima, Tomoko Shofuda, Daisuke Kanematsu, Ema Yoshioka, Yoshinori Kodama, Masayuki Mano, Kanji Mori, Koichi Ichimura, Yonehiro Kanemura

**Affiliations:** 1 Department of Neurosurgery, Osaka International Cancer Institute, Osaka, 541-8567 Japan; 2 Kansai Molecular Diagnosis Network for CNS Tumors, Osaka, 540-0006 Japan; 3 Department of Neurosurgery, Osaka University Graduate School of Medicine, Suita, 565-0871 Japan; 4 Division of Brain Tumor Translational Research, National Cancer Center Research Institute, Tokyo, 104-0045 Japan; 5 Center for Comprehensive Community Medicine, Center for Comprehensive Community Medicine, Faculty of Medicine, Saga University, Saga, 849-8501 Japan; 6 Department of Neurosurgery and Neuro-Oncology, National Cancer Center Hospital, Tokyo, 104-0045 Japan; 7 Department of Neurosurgery, Osaka City University Graduate School of Medicine, Osaka, 545-0051 Japan; 8 Department of Neurosurgery, Kindai University Faculty of Medicine, Sayama, 589-8511 Japan; 9 Department of Neurosurgery, National Hospital Organization Osaka National Hospital, Osaka, 540-0006 Japan; 10 Department of Neurosurgery, Kansai Medical University, Hirakata, 573-1191 Japan; 11 Department of Neurosurgery, Rinku General Medical Center, Izumisano, 598-8577 Japan; 12 Department of Neurosurgery, Kawachi General Hospital, Higashi-Osaka, 578-0954 Japan; 13 Department of Neurosurgery, Wakayama Medical University, Wakayama, 641-8509 Japan; 14 Department of Neurosurgery, Osaka City General Hospital, Osaka, 534-0021 Japan; 15 Department of Neurosurgery, Sakai City Medical Center, Sakai, 593-8304 Japan; 16 Division of Stem Cell Research, Institute for Clinical Research, Osaka National Hospital, National Hospital Organization, Osaka, 540-0006 Japan; 17 Division of Regenerative Medicine, Institute for Clinical Research, Osaka National Hospital, National Hospital Organization, Osaka, 540-0006 Japan; 18 Department of Pathology and Applied Neurobiology, Kyoto Prefectural University of Medicine, Kyoto, 602-8566 Japan; 19 Department of Central Laboratory and Surgical Pathology, Osaka National Hospital, National Hospital Organization, Osaka, 540-0006 Japan; 20 Department of Neurosurgery, Kansai Rosai Hospital, Amagasaki, 660-8511 Japan

## Abstract

Molecular biological characterization of tumors has become a pivotal procedure for glioma patient care. The aim of this study is to build conventional MRI-based radiomics model to predict genetic alterations within grade II/III gliomas attempting to implement lesion location information in the model to improve diagnostic accuracy. One-hundred and ninety-nine grade II/III gliomas patients were enrolled. Three molecular subtypes were identified: *IDH1/2*-mutant, *IDH1/2*-mutant with *TERT* promoter mutation, and *IDH-*wild type. A total of 109 radiomics features from 169 MRI datasets and location information from 199 datasets were extracted. Prediction modeling for genetic alteration was trained via LASSO regression for 111 datasets and validated by the remaining 58 datasets. *IDH* mutation was detected with an accuracy of 0.82 for the training set and 0.83 for the validation set without lesion location information. Diagnostic accuracy improved to 0.85 for the training set and 0.87 for the validation set when lesion location information was implemented. Diagnostic accuracy for predicting 3 molecular subtypes of grade II/III gliomas was 0.74 for the training set and 0.56 for the validation set with lesion location information implemented. Conventional MRI-based radiomics is one of the most promising strategies that may lead to a non-invasive diagnostic technique for molecular characterization of grade II/III gliomas.

## Introduction

Molecular biological characterization of tumors has become a pivotal procedure for glioma patient care. Identifying *IDH*1/2 mutation and 1p/19q chromosomal codeletion is now essential for the final pathological diagnosis of either an astrocytic or oligodendroglial tumor^1–4^. These molecular markers can be further utilized for predicting sensitivity of the tumor to chemotherapy and radiation and for further prognostication of the patient^3,4^. Unfortunately, current technology does not allow non-invasive detection of these molecular markers in gliomas, which necessitates direct tumor sampling via surgical removal of the tumor. Although aggressive removal of the tumor is thought to be beneficial for all of the patients suffering gliomas^5,6^, it is also true that some epidemiological researches have questioned the role of surgery in oligodendroglial tumors, as this type of tumor is extremely sensitive to chemotherapy and radiation^7,8^. Many previous studies have attempted to non-invasively predict *IDH*1/2 mutation^9–13^ or 1p/19q codeletion^14–16^ with various radiological modalities including conventional magnetic resonance imaging (MRI), advanced MRI such as diffusion and perfusion imaging, and molecular imaging using various nuclear medicine tracers. On the other hand, researchers in the field of radiology have become increasingly interested in multiple image texture features and lesion location analysis. Simultaneous multi-parametric quantification of radiological images is thought to enable image-based clustering of neoplasms. This newly emerging technique, named radiomics, may be useful for characterizing and identifying the tumor’s biological behaviors, which are defined by its inherent molecular biological nature. Thus, radiomics has the potential to non-invasively predict the molecular biological status of the tumor^17–22^. Preceding studies have attempted to detect genetic mutations within lung cancers or identify radiological features that correlate with glioblastoma patient prognosis^23,24^. These previous attempts mainly focused on performing texture analysis of the lesions under the hypothesis that different tumors with different genetic background will present their unique textures on radiological images. In the current report, the authors further pursuit the possibility of implementing lesion locations into magnetic resonance imaging (MRI) radiomics and devised a method for non-invasive molecular biological diagnosis of World Health Organization (WHO) grade II and III gliomas (grade II/III gliomas) using conventional MRI alone.

## Methods

### Patient cohort

This study was carried out in accordance with the principles of the Helsinki Declaration, and approval was obtained from the internal ethical review boards of Osaka International Cancer Institute (approval number: 1306055036), Osaka University Graduate School of Medicine (approval number: 13244), and all collaborative institutes. Written informed consent was obtained from all patients.

Inclusion criteria for the present study were as follows: 20 years of age or older, frozen or fresh tissue available for genomic analysis, MRI data including T2-weighted images available before the initial surgery, and local diagnosis of lower grade (WHO grade II-III) glioma based on the fourth edition of WHO Classification (WHO 2007)^25^. Finally, 199 cases from 11 institutions were eligible for analysis. Frozen or fresh tumor samples were obtained at the time of surgery. Tumor genomic DNA was extracted for genetic analysis. Clinical information of each case including age, sex, survival time, local diagnosis, and steroid use before image acquisition was collected from the medical records at each institution. Overall survival was determined as the time from the date of the initial surgery for diagnosis to the date of death or the last follow-up. The survival data were analyzed with the log-rank test and univariate and multivariate Cox regression analyses. Details are shown in Table S1.

### MRI

All MRIs analyzed in the present study were preoperatively acquired using either 1.5- or 3.0- MRI scanners according to the protocols in each institution. T2-weighted images were available in all cases. FLAIR and T1-weighted images were obtained in 179 and 194 cases, respectively. T1-weighted images after gadolinium enhancement were also available in 194 cases. Details are shown in Supplementary dataset.

### Diagnosis and central pathology

All cases were subjected to central pathology review by senior neuropathologists. Integrated diagnosis was made based on microscopic histological diagnosis and the status of *IDH*1/2 and 1p/19q copy number in compliance with the CNS WHO2016^26^.

### Genetic analysis

Genetic analyses were performed in two laboratories: the Osaka National Hospital (ONH), Osaka, Japan and the National Cancer Center Research Institute (NCC), Tokyo, Japan. Hotspot mutations of *IDH*1/2 (codon 132 of *IDH*1 and codon 172 of *IDH*2) and the *TERT* promoter (termed C228 and C250) were assessed by Sanger sequencing and/or pyrosequencing at either lab. 1p/19q copy number status was analyzed with multiplex ligation-dependent probe amplification in a unified workflow at either lab. Detailed information of the genetic analysis for *IDH*1/2, *TERT*, and 1p/19q can be found in a previous publication^27^. The methylation status of the *MGMT* promoter was analyzed and assessed by qPCR at ONH or by pyrosequencing after bisulfite modification at NCC. Detailed information of pyrosequencing and qPCR for *MGMT* was described previously^27,28^.

### Radiomics

Radiomic analyses (radiomics) were conducted by in-house-developed image analyzing software in combination with Oxford Centre for Functional MRI of the Brain (FMRIB) Linear Image Registration Tool (FLIRT) provided by FMRIB Software Library (FSL)^29–31^. The in-house software was developed in Matlab (Mathworks, Natick, MA), and seamless data transfer was carried out between Matlab-based in-house software and FSL via FSL integration into Matlab. All Digital Imaging and Communications in Medicine format images were first converted to Neuroimaging Informatics Technology Initiative (NIfTI) format using MRIconvert (University of Oregon Lewis Center for Neuroimaging: http://lcni.uoregon.edu/jolinda/MRIConvert/), followed by 256 gray-scale level conversion. For, non-contrast T1-weighted, gadolinium contrast-enhanced T1-weighted, and FLAIR images, voxels that were in the top 0.1% in intensity were deleted as they were mainly high signal noises, and the remaining 99.9% were reallocated in 256 gray-scale. For T2-weighted images, 100% of the data range was reallocated in 256 gray-scale. This procedure was necessary for intensity normalization across all images acquired by different MR scanners. Furthermore, T2Edge images were constructed by applying a Prewitt filter to T2-weighted images. Gdzscore images were also constructed by performing a voxel-wise contrast enhancement calculation using non-contrast and gadolinium contrast-enhanced T1-weighted images. Detailed methods can be found in Table S1. Tumors were delineated by manually tracing high-intensity lesions on T2-weighted images in three dimensions by an experienced surgical neuro-oncologist (M.K.).

First, NIfTI data of the T2-weighted images were registered to a 1.0-mm isotropic, high-resolution T1-weighted brain atlas provided by the MNI152 using a mutual information algorithm with a 12-degree of freedom transformation with FSL-FLIRT. This procedure was necessary to perform lesion mapping of the tumors in standard MNI152 space. Next, all different image sequences obtained from a single subject were co-registered to each other using FSL-FLIRT to obtain transformation matrices of different image sequences. Three-dimensional lesion VOIs modeled on T2-weighted images as mentioned-above were further used for radiomics and registered to different MR sequences including MNI152 using the transformation matrices calculated by FSL-FLIRT. VOIs registered to MNI152 were used for location mapping. Three different aspects of the tumor were measured, i.e., histogram-based first-order texture, shape of the tumor, and location (Table S1).

### Statistical analysis and predictive modeling

Statistical analysis was performed by M.K. and a biostatistician (A.K.). Survival analysis was performed with the Kaplan-Meier method with multiple group comparison using proportional hazard. A correlation matrix of the radiomic features was calculated and visualized. These analyses were performed by JMP Pro ver.13 (SAS, Cary, NC). Random permutation analysis was performed to test the statistical significance of differences in lesion occurrence between the three different molecular subtypes of grade II/III gliomas in this cohort, similar to the method described previously^32–34^. In short, a voxel-wise two-tailed Fisher’ exact test for a 3 × 2 contingency table, which compared three different molecular subtypes of grade II/III gliomas and lesion-positive with lesion-negative, was conducted with all voxels containing at least one lesion occurrence; the *p* value threshold was set at 0.05. A cluster-based permutation correction was performed for multiple comparison correction, in which the statistical testing was repeated 500 times with grade II/III gliomas lesions randomly reassigned to three different molecular subtypes of grade II/III gliomas.

Predictive modeling was performed based on least absolute shrinkage and selection operator (LASSO) method to select features that were most significant to build predictive models for identifying genetic mutations of the tumor. λ which is the tuning parameter for LASSO was selected for which the cross-validation error is smallest (λ_min). The final predictive models were refit using the significant components chosen by LASSO and λ_min. Calculation was performed on R using the Glmnet package using 109 radiomic features and 169 datasets, which had T1-, T2-weighted, FLAIR, and gadolinium-enhanced T1-weighted images available. These 169 datasets were divided randomly but balancing institutions into 111 training datasets and 58 validation datasets. The constructed predictive model using the training datasets were then applied to the validation datasets, which was randomly selected from the pooled 169 datasets to examine the validity of the constructed model. The codes and data used for analysis are provided as supplementary materials. The R code for Lasso regression is available in Table S2 and the data used for this code is provided as dataset171101Train.csv and dataset171101Validation.csv.

### Data and materials availability

Raw analyzed data are available in Supplementary dataset.

## Results

### Cohort validation

**Figure 1:**
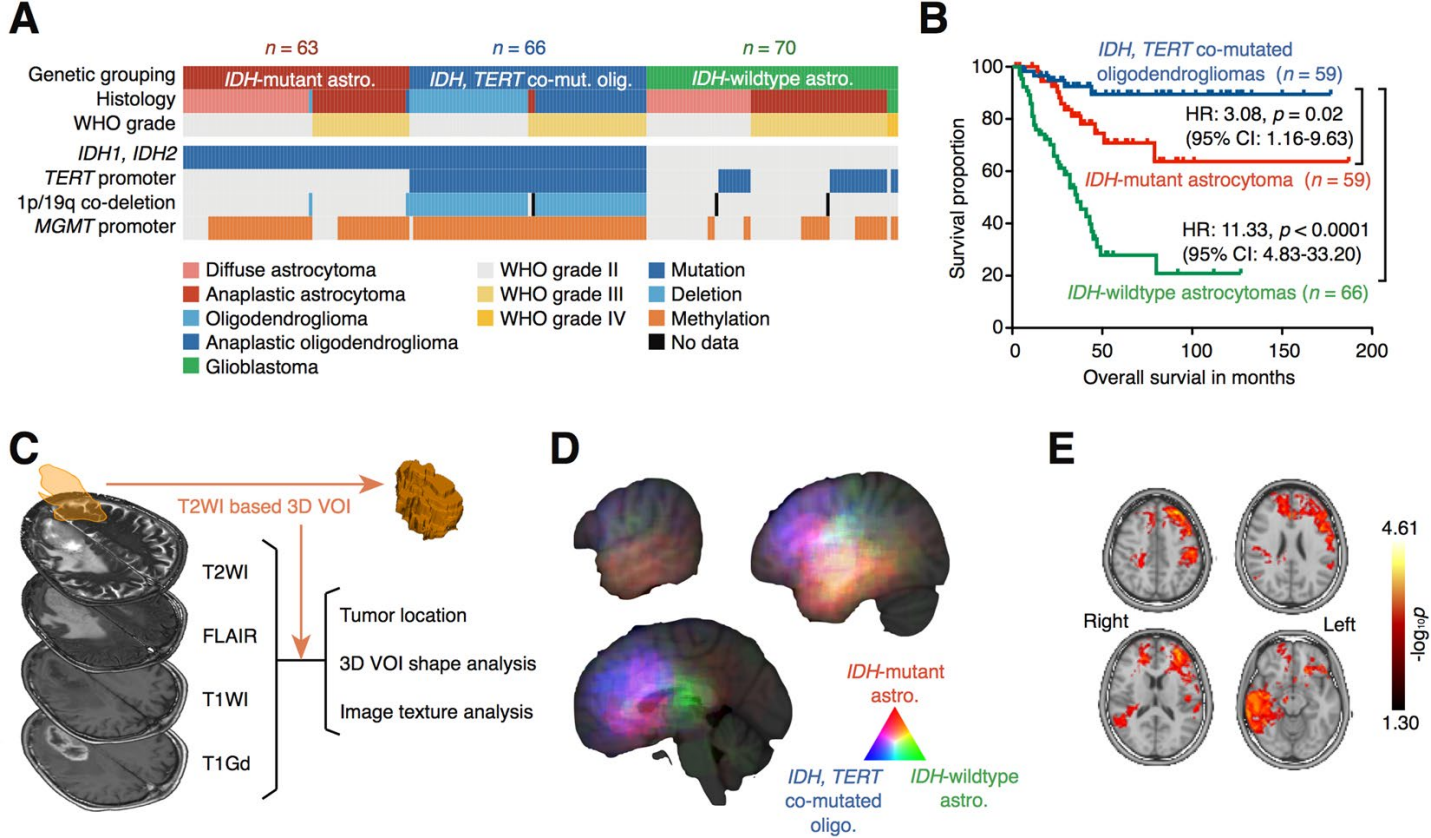
Overview of the analyzed cohort and image analyses (radiomics) with voxel-based lesion mapping. (**A**) Landscape of genetic and pathological lesions of the cohort is presented. Genetic status and central pathological reviews are shown by color as indicated. (**B**) Kaplan-Meier curves for the three types of tumors from the analyzed cohort are presented. Hazard ratios (HR) were calculated by considering the *IDH, TERT* co-mutated oligo. group as a reference. (**C**) Overview of radiomics is presented. Detailed methods for analysis are descried in Table S2. (**D**) Color-coded voxel-wise lesion mapping of the three different molecular subtypes of this cohort. Frequency of the locations of the brain affected by each molecular subtype are color coded as indicated. Detailed mapping is provided in Fig. S1–3. (**E**) Random permutation analysis shows locations that exhibited statistically significant differences in spatial distribution of the lesion among the three different molecular subtypes of the analyzed cohort. Detailed mapping is provided in Fig. S4.

A total of 199 in-house diagnosed grade II/III gliomas were retrospectively collected and molecular subtype was determined following surgical removal of the tumor. *IDH*1/2 and *TERT* promoter mutations were used for molecular subtype classification. One hundred twenty-nine cases (64.8% of all cases) harbored the hotspot mutation in *IDH*1/2. *TERT* promoter mutations were observed in 93 cases (46.7% of all cases). 1p/19q status was obtained for all but three cases. Sixty-six cases (33.7%, 66/196) exhibited 1p/19q codeletion. 1p/19q status was available for all but one case in the group with mutations in both *IDH*1/2 and *TERT*, and the great majority of this group overlapped with 1p/19q-codeleted tumors. Three types of molecular subtypes were identified: *IDH1/2*-mutant (*IDH*-mutant astrocytomas), *IDH1/2*-mutant with *TERT* promoter mutation (*IDH, TERT* co-mutated oligodendrogliomas), and *IDH-*wild type (*IDH-*wildtype astrocytomas). *TERT* promoter mutation was preferably used over 1p/19q codeletion for molecular subtyping of the tumor according to previous reports that confirmed that this genetic mutation is prognostic^2,27^. Central pathological examination suggested that three cases that were diagnosed in-house as WHO grade III could possibly be WHO grade IV (Fig. 1A and Supplementary dataset).

The prognosis of each group was consistent with investigation of other previous large cohorts, thus validating further use of this cohort as a representative grade II/III gliomas population. *IDH, TERT* co-mutated oligodendrogliomas showed the most favorable prognosis followed by *IDH*-mutant astrocytomas only. *IDH-*wildtype astrocytomas had the worst prognosis (Fig. 1B). Radiomics was performed with conventional MRI of this grade II/III gliomas cohort. Lesions were segmented in three dimensions based on T2-weighted images, and locations, textures, and shapes of the lesions were analyzed (Fig. 1C and Table S1).

### Location analysis of different molecular subtypes for grade II/III gliomas

Voxels of interest (VOIs) were registered to the standard anatomical MR atlas, Montreal Neurological Institute (MNI152), and the lesions were mapped on the atlas. When the laterality of the lesions was ignored and mapped on MNI152, the three subtypes of grade II/III gliomas showed different distinct locations. *IDH*-mutant astrocytomas preferably involved the temporal lobe and the frontal lobe to some extent. *IDH, TERT* co-mutated oligodendrogliomas preferably involved the frontal lobe. We also noted that the medial frontal cortex was a unique location for this type of tumor. On the other hand, *IDH-*wildtype astrocytomas occupied the parietal lobe and to some extent the temporal lobe with little frontal lobe involvement (Fig. 1D, Fig. S1–3). This tendency was maintained even when the laterality of the lesions was preserved during location analysis via random permutation analysis, and we confirmed that the frontal and temporal lobes were locations that unevenly involved the different molecular subtypes of grade II/III gliomas (Fig. 1E and S4). Locations of the VOIs were further quantified by calculating the occupancy ratio of the VOIs on two different brain segmentation atlases, namely the “MNI structural atlas”^35^ and “Harvard-Oxford cortical structural atlas”^36^ (Table S1). The “MNI structural atlas” segments the brain in 10 different anatomical locations including the deep white matter. The “Harvard-Oxford cortical structural atlas”, on the other hand, segments the cortical anatomy into 49 locations, which atlas further looks into cortical anatomy more in detail compared to “MNI structural atlas”, but does not provide information on the deep white matter.

### Radiomics of grade II/III gliomas

**Figure 2:**
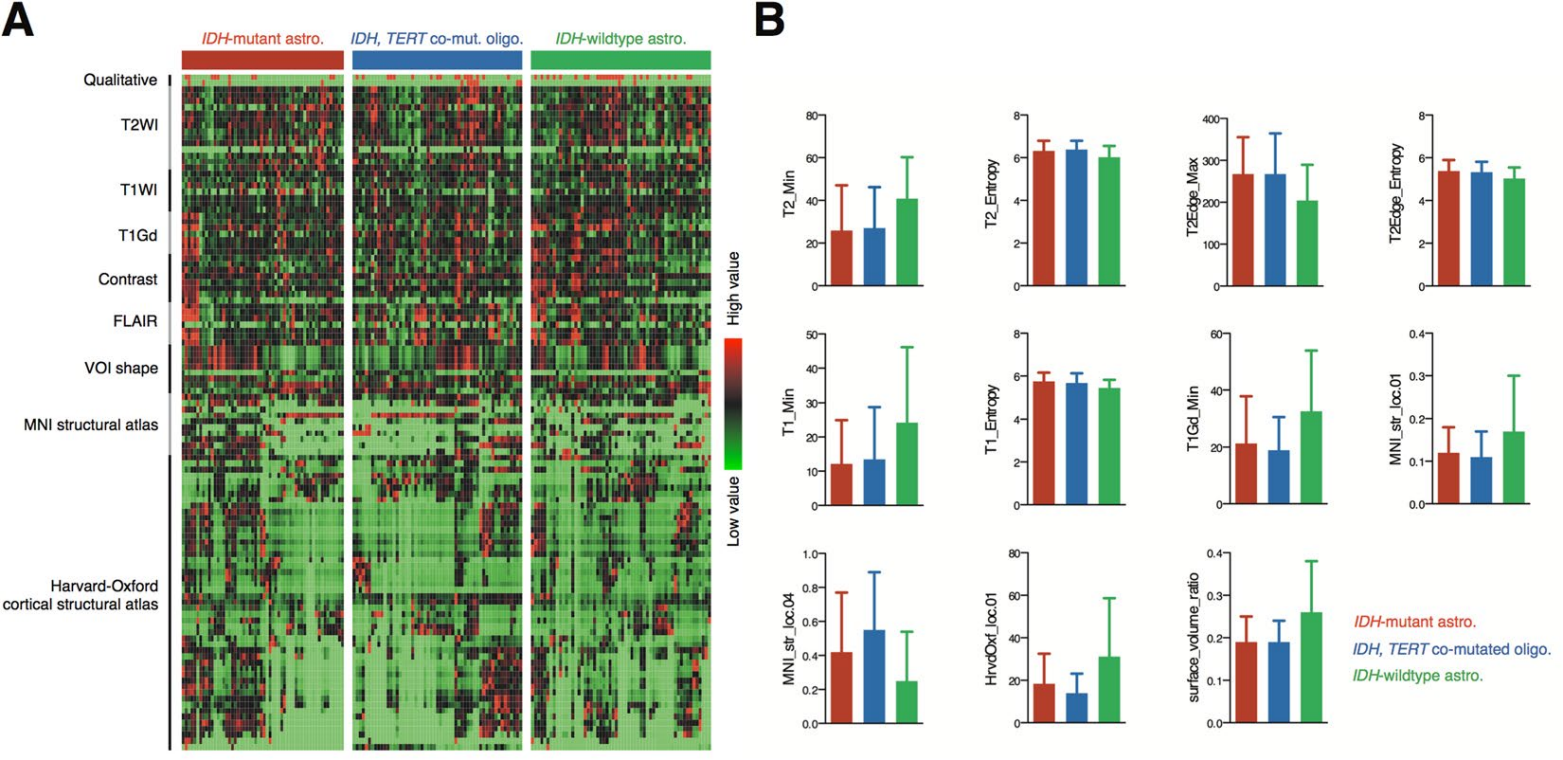
Radiomics measurements. (**A**) Overview of radiomics of the current grade II/III gliomas cohort is shown. Major components of the analysis are listed on the left side of the figure in rows. (**B**) Measurements with an extremely low *p* value (<0.001) with one-way ANOVA are presented. Each colored bar represents the different molecular subtype of the tumor as in (**A**). Data are presented as the mean ± standard deviation. More details can be found in Tables S1 and 3.

**Figure 3:**
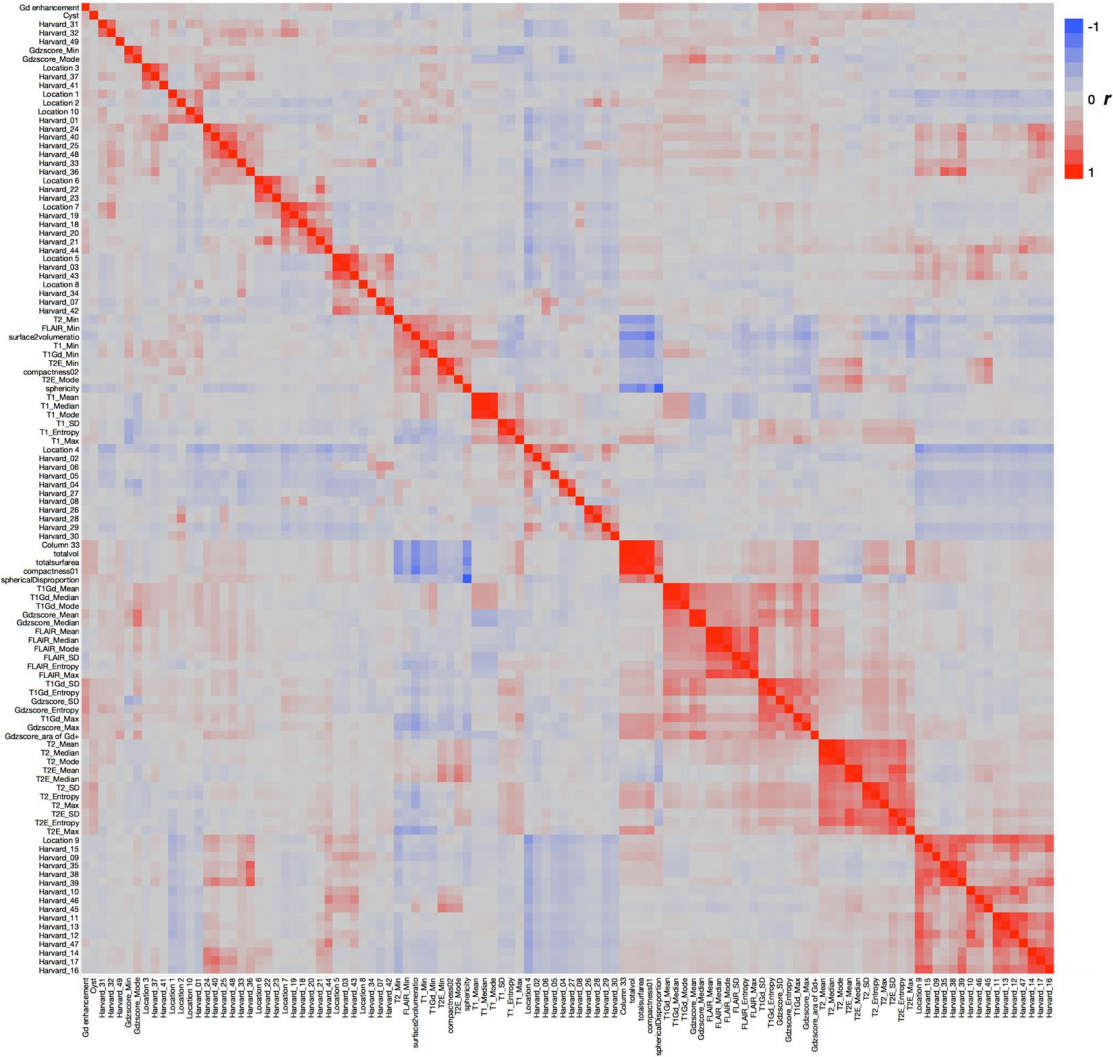
Correlation matrix heat map of radiomics. The correlation matrix of all radiomic parameters is visualized in a heat map. The magnitude of the correlation is indicated in the color bar.

A total of 109 radiomic features were quantified and collected (Fig. 2A, Table S3). A correlation metric of the 109 radiomic features is shown in Fig. 3 and indicates little redundancy. We assessed 169 complete datasets that included T1-weighted, T2-weighted, fluid attenuated inversion recovery (FLAIR), and gadolinium contrast-enhanced images. Eleven features were significantly different among the three molecular subtypes of grade II/III gliomas (*p* < 0.001, one-way ANOVA, Fig. 2B), 13 features were significantly different with a *p* value ranging from 0.001 to less than 0.01, and 15 features were significantly different with a *p* value ranging from 0.01 to less than 0.05 (Table S3). Of the 11 features with a *p* value of less than 0.001, four were related to T2-weighted images, two to T1-weighted images, one to gadolinium contrast-enhanced images, another to the VOI shape, and three to location. For the three location-related parameters, MNI_str_loc.01 and HrvdOxf_loc.01 are segments of the brain that both correspond to the white matter, the values of which are assigned as “0” in the original atlas. *IDH-*wildtype astrocytomas showed higher occupancy in the white matter than other groups, as both MNI_str_loc.01 and HrvdOxf_loc.01 were highest for *IDH-*wildtype astrocytomas. MNI_str_loc.04, with an original value of “3,” corresponds to the frontal lobe, which showed the lowest value in *IDH-*wildtype astrocytomas.

### Predicting IDH mutation status of grade II/III gliomas via lesion location implemented radiomics

**Figure 4:**
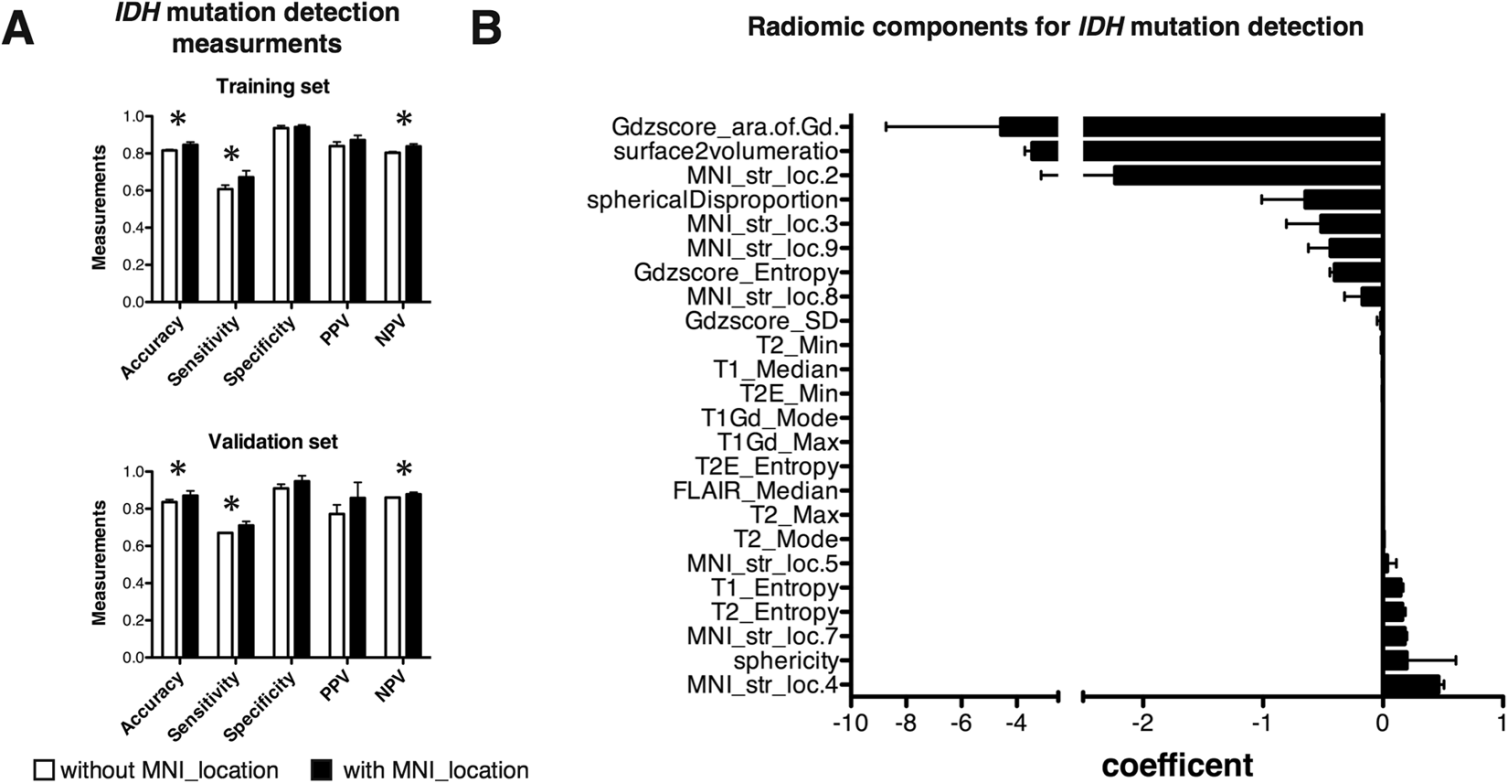
*IDH* mutation predictive modeling with and without lesion location information radiomics. (**A**) Overall diagnostic accuracy for the training set and the validation set is shown. Diagnostic accuracy without lesion location information was as high as 0.82 for the training set and 0.83 for the validation set. This accuracy further improved to 0.85 (*p* = 0.01) and 0.87 (*p* = 0.04) respectively by including lesion location info. Sensitivity and negative predictive value also improved significantly by implementing lesion location information in predictive modeling (*p < 0.05). (**B**) Radiomic components significant for predictive modeling is shown. Frontal lobe tumor involvement (MNI_str_loc.04) was one of the most significant features for being the tumor to be *IDH* mutated, while the magnitude of contrast enhancement (Gdzscore_ara.of.Gd.) was for *IDH-*wt. Averages and standard deviations of 5 repetitive analyses are shown for both (**A)** and (**B**). PPV stands for “positive predictive value” and NPP for “negative predictive value”.

A discrimination model for predicting *IDH* mutation of grade II/III gliomas was constructed using a LASSO regression algorithm. Texture information with or without lesion location information was utilized for predictive model construction. Lesion location information deriving from the “MNI structural atlas” was adopted for this attempt, as location information including that of the deep white matter is necessary. Diagnostic accuracy without lesion location information was as high as 0.82 for the training set and 0.83 for the validation set (Fig. 4A). This accuracy further improved to 0.85 (*p* = 0.01) and 0.87 (*p = *0.04) respectively by including lesion location information (Fig. 4A). Sensitivity and negative predictive value also improved significantly by implementing lesion location information in predictive modeling. Significant radiomic components that were selected for predictive modeling is shown in Fig. 4B. Again, frontal lobe tumor involvement (MNI_str_loc.04) was one of the most significant features for being the tumor to be *IDH*-mutated, while the magnitude of contrast enhancement (Gdzscore_ara.of.Gd.) was for *IDH-*wildtype.

### Predicting 3 molecular subtypes of grade II/III gliomas via lesion location implemented radiomics

**Figure 5:**
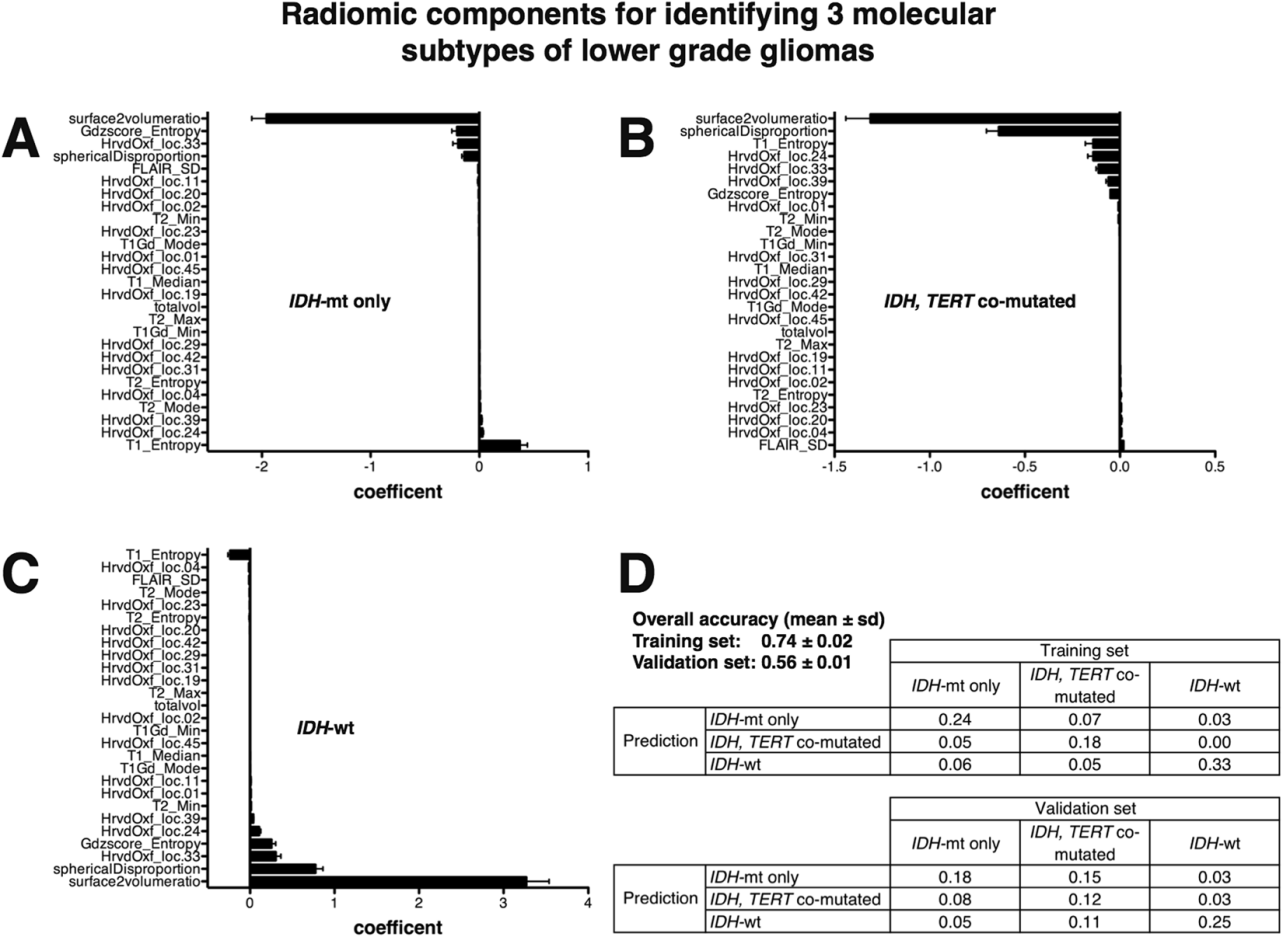
Radiomics predictive modeling for 3 genetic subtypes of grade II/III gliomas. (**A~C**) Significant radiomic components for predicting modeling enabling diagnosing 3 genetic subtypes of grade II/III gliomas are shown. (**D**) Overall diagnostic accuracies were 0.74 for the training set and 0.56 for the validation set. The confusion matrix is also shown to elucidate correctly and miss classified components. Averages and standard deviations of 5 repetitive analyses are shown for all the data.

A discrimination model for predicting 3 molecular subtypes of grade II/III gliomas was constructed using a LASSO regression algorithm. Texture information with lesion location information was utilized for predictive model construction. Lesion location information deriving from the “Harvard-Oxford cortical structural atlas” was adopted for this attempt contrasting the above-mentioned attempt to predict *IDH* mutation. This was because more detailed information was thought necessary to discriminate *IDH*-mutant astrocytomas from *IDH* and *TERT* co-mutated oligodendrogliomas, however, losing location information related to the deep white matter by abandoning “MNI structural atlas”. Using both information was avoided as some part of the information may be redundant. Many location related radiomic components along with texture information were considered significant for predictive modeling (Fig. 5A–C). Overall diagnostic accuracy was 0.74 for the training set and 0.56 for the validation set (Fig. 5D) while the expected value would be 0.33 as this is a 3-group classification problem.

## Discussion

This investigation is one of the few that tested the hypothesis that genetic alterations in grade II/III gliomas can be distinguished with images obtained by conventional MRI and that these images can be predictive for determining molecular subtypes of grade II/III gliomas. Genetic characterization of grade II/III gliomas has become one of the most crucial practices in the management of this neoplasm. Genetic analyses of grade II/III gliomas using large-scale cohorts revealed several key molecular alterations in grade II/III gliomas that are biologically and clinically significant^1–4^. Such mutations include *IDH*1/2 mutation, 1p/19q codeletion, and *TERT* promoter mutation. *IDH*1/2-mutant tumors either present with or without 1p/19q codeletion. 1p/19q codeletion usually overlaps with *TERT* promoter mutation, which was reproduced in the current cohort^1,2^. These three genetic alterations are considered “truncal mutations” that occur in one of the earliest phases of oncogenesis^1^. From a clinical perspective, these mutations are predictive and prognostic for grade II/III glioma patients. While extent of tumor resection and residual tumor volume has been shown to be significant prognostic factors for low grade gliomas^5–7,37,38^, *IDH*1/2-mutant and 1p/19q codeleted tumors are extremely sensitive to chemotherapy and radiation and it was reported that extent of resection was not prognostic for this type of tumor^7,8^, which oncological feature is less prominent in *IDH*1/2-mutant and 1p/19q non-codeleted gliomas^5–7,38^. On the other hand, although *IDH-*wild-type tumors may be diagnosed as WHO grade II or III, they are associated with an extremely poor prognosis, similar to that of glioblastoma^1,2^. These genetic alterations are detected by direct examination of the resected tumor specimen using immunohistochemistry, fluorescence *in situ* hybridization, and DNA sequencing. A non-invasive method of identifying a subset of patients who could benefit from radiation and chemotherapy rather than from surgical removal would reduce the burden and the unnecessary risk to those patients. Several attempts have been made to achieve this objective in the past. For example, tumors with *IDH*1/2 mutation accumulate 2-hydroxyglutarate (2-HG) within the tumor, and magnetic resonance spectroscopy (MRS) is thought to be a promising technique to non-invasively detect 2-HG and thus suggest *IDH*1/2 mutation of the tumor. Past MRS investigations were able to image 2-HG presence^12,13^. This technique, however, is still not available in routine clinical imaging. Another modality that is gaining interest is perfusion MRI. One study analyzed 73 dynamic susceptibility contrast-enhanced MRIs and showed that *IDH*1/2-mutant WHO grade II and III tumors tend to present with lower regional cerebral blood volume than *IDH-*wildtype tumors^9^, with another similar investigation reporting the same trend^10^.

As these “advanced” MRI techniques depend on technologies for which the optimum settings for various parameters are still undetermined, a few studies have been conducted to develop a method to predict the molecular status of gliomas by analyzing standard MRI. These studies have clearly suggested that the inherent nature of these tumors including their molecular characteristics can be detected on radiological images. *IDH*1/2-mutant tumors tend to occur in the frontal or temporal lobe, and this information is valuable for predicting whether a lesion that appears to be a WHO grade II or III glioma harbors this mutation^39–41^. Furthermore, textures of the lesion are also informative for predicting the molecular status of grade II/III gliomas^14,42^. A sharp tumor border and heterogeneity of the lesion provide an image surrogate for identification of 1p/19q codeletion status^14^ or *IDH*1/2 mutation^42^.

Recently more advanced image analyses have been reported to predict genetic alterations in grade II/III gliomas^19,20,43,44^. These analyses have taken advantage of radiomics and machine learning algorithms to comprehensively and quantitatively analyze the radiological images of each tumor and use those measurements to construct a predictive model to identify the genetic status or determine prognostic image biomarkers of the neoplasm. Other types of cancer such as glioblastoma, breast cancer, and lung cancer have been extensively explored to assess the usefulness of this novel emerging technique. Some image biomarkers may now be informative for predicting *MGMT* promoter methylation status or prognosis of glioblastoma^22,45,46^, for prognosis for breast cancer^47,48^, and for predicting *EGFR* mutation status or prognosis of non-small cell lung cancer^23,24^. The current investigation is one of the few that focused on radiomics and grade II/III gliomas molecular status. Although similar attempts have been made to identify *IDH*1/2 mutation and 1p/19q codeletion via radiomics, most of them included glioblastomas within the analyzed cohort, and only several of them focused on grade II/III gliomas^20,43,44^. The preset research is also unique in that it took an approach to implement lesion location information into texture based radiomics as tumor location was considered a key feature of grade II/III gliomas that illustrates its inherent biological characteristics. The problem of handing location information was solved by performing lesion mapping on the standard MNI152 space and utilizing publicly available brain segmentation templates, which procedure converts spatial information into numerical data suitable for further modeling. By performing this analysis, it was clearly shown that *IDH-*wildtype astrocytoms preferentially localized in the white matter, and *IDH*1/2-mutant astrocytomas in the frontal, insular, and temporal lobe. We also noted that the medial frontal cortex was a location specific for *IDH*1/2-mutant with *TERT* promoter mutant oligodendrogliomas (Fig. 1D). Lesion location information was crucial for constructing a predictive model to identify molecular subtypes of the tumor (Figs 4 and 5). As a matter of fact, implementing lesion location information significantly improved prediction accuracy of *IDH* mutation status of grade II/III gliomas from 0.83 to 0.87 in the validation set. Other texture or histogram features measured from different MR sequences were also valuable for constructing the model (Figs 4 and 5), which suggests that all images analyzed, i.e., T1-, T2-weighted, FLAIR, and gadolinium contrast-enhanced images, represent different aspects of the tumor. In the end, the build model was able to achieve an accuracy of 0.85 for the training and 0.87 for the validation set in terms of predicting *IDH* mutation status of grade II/III gliomas, which is comparable or superior to previous reports as the current report performed the accuracy of the built model using a validation dataset.

On the other hand, although prediction accuracy of 3 molecular subtypes of grade II/III gliomas (0.74 for the training set and 0.56 for the validation set) was higher than the expected value (0.33), the modest prediction accuracy illustrates the need for implementing second-order texture analysis or adding more MR sequences such as diffusion and perfusion images to improve prediction accuracy. Comparison with other radiomic analytic tools will also be required for cross-validation among different radiomic algorithms.

## Conclusions

The authors devised an MRI lesion location implemented radiomics-based predictive model for determining molecular subtypes of grade II/III gliomas. *IDH* mutation can be predicted with an accuracy of 0.85 to 0.87, which accuracy improved by implementing lesion location information. Prediction accuracy of 3 molecular subtypes of grade II/III gliomas ranged from 0.56 to 0.74, requiring more tuning for clinical application. Although validation and more extensive testing are necessary to perfect this technology for real clinical use, radiomics-based non-invasive prediction of molecular subtypes of grade II/III gliomas is a promising approach to personalized medicine in the field of glioma treatment, which technology could highly impact treatment workflow of this disease including tumor genetic information-implemented presurgical planning of gliomas.

## Electronic supplementary material

Supplementary information

Supplementary dataset

dataset171101Train.csv

dataset171101Validation.csv
