# Supplementary material for: Lesion location implemented magnetic resonance imaging radiomics for predicting IDH and TERT promoter mutations in grade II/III gliomas
Source: Sci Rep. 2018 Aug 6;8:11773. doi: 10.1038/s41598-018-30273-4 (PMC6078954; doi:10.1038/s41598-018-30273-4)

Supplementary information

Lesion location implemented magnetic resonance imaging radiomics for predicting *IDH* and *TERT* promoter mutations in grade II/III gliomas

Hideyuki Arita[1,2,3,4], Manabu Kinoshita[1,2*], Atsushi Kawaguchi[5], Masamichi Takahashi[6], Yoshitaka Narita[6], Yuzo Terakawa[2,7], Naohiro Tsuyuguchi[2,7,8], Yoshiko Okita[2,9], Masahiro Nonaka[2,10], Shusuke Moriuchi[2,9,11], Masatoshi Takagaki[2,12], Yasunori Fujimoto[2,3], Junya Fukai[2,13], Shuichi Izumoto[2,8], Kenichi Ishibashi[2,14], Yoshikazu Nakajima[2,15], Tomoko Shofuda[2,16], Daisuke Kanematsu[2,17], Ema Yoshioka[2,17], Yoshinori Kodama[2,18,19], Masayuki Mano[2,19] Kanji Mori[2,20], Koichi Ichimura[4], and Yonehiro Kanemura[2,17]

1 Department of Neurosurgery, Osaka International Cancer Institute, Osaka 541-8567, Japan.

2 Kansai Molecular Diagnosis Network for CNS Tumors, Osaka 540-0006, Japan.

3 Department of Neurosurgery, Osaka University Graduate School of Medicine, Suita 565-0871, Japan.

4 Division of Brain Tumor Translational Research, National Cancer Center Research Institute, Tokyo 104-0045, Japan.

5 Center for Comprehensive Community Medicine, Center for Comprehensive Community Medicine, Faculty of Medicine, Saga University, Saga 849-8501, Japan.

6 Department of Neurosurgery and Neuro-Oncology, National Cancer Center Hospital, Tokyo 104-0045, Japan.

7 Department of Neurosurgery, Osaka City University Graduate School of Medicine, Osaka 545-0051, Japan.

8 Department of Neurosurgery, Kindai University Faculty of Medicine, Sayama 589-8511, Japan.

9 Department of Neurosurgery, National Hospital Organization Osaka National Hospital, Osaka 540-0006, Japan.

10 Department of Neurosurgery, Kansai Medical University, Hirakata 573-1191, Japan.

11 Department of Neurosurgery, Rinku General Medical Center, Izumisano 598-8577, Japan

12 Department of Neurosurgery, Kawachi General Hospital, Higashi-Osaka 578-0954, Japan.

13 Department of Neurosurgery, Wakayama Medical University, Wakayama 641-8509, Japan.

14 Department of Neurosurgery, Osaka City General Hospital, Osaka 534-0021, Japan.

[15] Department of Neurosurgery, Sakai City Medical Center, Sakai 593-8304, Japan

[16] Division of Stem Cell Research, Institute for Clinical Research, Osaka National Hospital, National Hospital Organization, Osaka 540-0006, Japan.

[17] Division of Regenerative Medicine, Institute for Clinical Research, Osaka National Hospital, National Hospital Organization, Osaka 540-0006, Japan.

[18] Department of Pathology and Applied Neurobiology, Kyoto Prefectural University of Medicine, Kyoto 602-8566, Japan.

[19] Department of Central Laboratory and Surgical Pathology, Osaka National Hospital, National Hospital Organization, 540-0006, Japan

[20] Department of Neurosurgery, Kansai Rosai Hospital, Amagasaki 660-8511, Japan.

***To whom correspondence should be addressed:** Manabu Kinoshita M.D., Ph.D.

Department of Neurosurgery, Osaka International Cancer Institute

3-1-69 Otemae, Chuo-ku, Osaka 541-8567 Japan

TEL: +81-6-6945-1181 FAX: +81-6-6945-1900

Email: mail@manabukinoshita.com

Supplementary Materials

Table S1. Measured parameters for radiomics.

Table S2. R source codes for Lasso regression.

Table S3. Statistical analysis of measured radiomics parameters.

Fig. S1. Location mapping on MNI152 in the axial plane.

Fig. S2. Location mapping on MNI152 in the coronal plane.

Fig. S3. Location mapping on MNI152 in the sagittal plane.

Fig. S4. Random permutation analysis mapping in the axial plane.

Table S1

Radiomic designation code	Contents of values
Histogram and texture parameters	
T2_Mean	Mean of VOI in 256-leveled T2-weighted image.
T2_SD	Standard deviation of VOI in 256-leveled T2-weighted image.
T2_Max	Maximum of VOI in 256-leveled T2-weighted image.
T2_Min	Minimum of VOI in 256-leveled T2-weighted image.
T2_Median	Median of VOI in 256-leveled T2-weighted image.
T2_Mode	Mode of VOI in 256-leveled T2-weighted image.
T2_Entropy	Entropy[1)] of VOI in 256-leveled T2-weighted image.
T2Edge_Mean	Mean of the rim of VOI in Prewitt filtered[2)] 256-leveled T2-weighted image.
T2Edge_SD	Standard deviation of the rim of VOI in Prewitt filtered[2)] 256-leveled T2-weighted image.
T2Edge_Max	Maximum of the rim of VOI in Prewitt filtered[2)] 256-leveled T2-weighted image.
T2Edge_Min	Minimum of the rim of VOI in Prewitt filtered[2)] 256-leveled T2-weighted image.
T2Edge_Median	Median of the rim of VOI in Prewitt filtered[2)] 256-leveled T2-weighted image.
T2Edge_Mode	Mode of the rim of VOI in Prewitt filtered[2)] 256-leveled T2-weighted image.
T2Edge_Entropy	Entropy[1)] of the rim of VOI in Prewitt filtered[2)] 256-leveled T2-weighted image.
T1_Mean	Mean of VOI in 256-leveled T1-weighted image.
T1_SD	Standard deviation of VOI in 256-leveled T1-weighted image.
T1_Max	Maximum of VOI in 256-leveled T1-weighted image.
T1_Min	Minimum of VOI in 256-leveled T1-weighted image.
T1_Median	Median of VOI in 256-leveled T1-weighted image.
T1_Mode	Mode of VOI in 256-leveled T1-weighted image.
T1_Entropy	Entropy[1)] of VOI in 256-leveled T1-weighted image.
T1Gd_Mean	Mean of VOI in 256-leveled Gadolinium enhanced T1-weighted image.

T1Gd_SD	Standard deviation of VOI in 256-leveled Gadolinium enhanced T1-weighted image.
T1Gd_Max	Maximum of VOI in 256-leveled Gadolinium enhanced T1-weighted image.
T1Gd_Min	Minimum of VOI in 256-leveled Gadolinium enhanced T1-weighted image.
T1Gd_Median	Median of VOI in 256-leveled Gadolinium enhanced T1-weighted image.
T1Gd_Mode	Mode of VOI in 256-leveled Gadolinium enhanced T1-weighted image.
T1Gd_Entropy	Entropy[1)] of VOI in 256-leveled Gadolinium enhanced T1-weighted image.
Gdzscore_Mean	Mean of VOI in Gdzscore[3)] image.
Gdzscore_SD	Standard deviation of VOI in Gdzscore[3)] image.
Gdzscore_Max	Maximum of VOI in Gdzscore[3)] image.
Gdzscore_Min	Minimum of VOI in Gdzscore[3)] image.
Gdzscore_Median	Median of VOI in Gdzscore[3)] image.
Gdzscore_Mode	Mode of VOI in Gdzscore[3)] image.
Gdzscore_Entropy	Entropy[1)] of VOI in Gdzscore[3)] image.
Gdzscore_area_of_Gd	Ratio of volume with Gdzscore>2.0 within the VOI.
FLAIR_Mean	Mean of VOI in 256-leveled FLAIR.
FLAIR_SD	Standard deviation of VOI in 256-leveled FLAIR.
FLAIR_Max	Maximum of VOI in 256-leveled FLAIR.
FLAIR_Min	Minimum of VOI in 256-leveled FLAIR.
FLAIR_Median	Median of VOI in 256-leveled FLAIR.
FLAIR_Mode	Mode of VOI in 256-leveled FLAIR.
FLAIR_Entropy	Entropy[1)] of VOI in 256-leveled FLAIR.
Shape related parameters	
totalsurfarea	Total surface area (A) of the VOI.
totalvol	Total volume (V) of the VOI.
compactness01	Value calculated by the following equation; $compactness1 = \frac{V}{\sqrt{\pi} * A^{\frac{2}{3}}}$
compactness02	Value calculated by the following equation; $compactness2 = 36\pi \frac{A^2}{V^3}$
spherical_disproportion	Value calculated by the following equation; $spherical\_disproportion = \frac{A}{4\pi * R^2} = \frac{A}{(6\sqrt{\pi} * V)^{\frac{2}{3}}}$
sphericity	Value calculated by the following equation; $sphericity = \frac{(6\pi^2 V)^{\frac{2}{3}}}{A}$
surface_volume_ratio	Value calculated by the following equation;

	$surface\_volume\_ratio = \frac{A}{V}$
Location related parameters	
MNI_str_loc.01	Occupancy rate of area "0" of the MNI structural atlas within the VOI. This area represents white matter.
MNI_str_loc.02	Occupancy rate of area "1" of the MNI structural atlas within the VOI. This area represents lateral ventricles.
MNI_str_loc.03	Occupancy rate of area "2" of the MNI structural atlas within the VOI. This area represents the cerebrum.
MNI_str_loc.04	Occupancy rate of area "3" of the MNI structural atlas within the VOI. This area represents the frontal lobe.
MNI_str_loc.05	Occupancy rate of area "4" of the MNI structural atlas within the VOI. This area represents the insular lobe.
MNI_str_loc.06	Occupancy rate of area "5" of the MNI structural atlas within the VOI. This area represents the occipital lobe.
MNI_str_loc.07	Occupancy rate of area "6" of the MNI structural atlas within the VOI. This area represents the parietal lobe.
MNI_str_loc.08	Occupancy rate of area "7" of the MNI structural atlas within the VOI. This area represents the basal ganglia.
MNI_str_loc.09	Occupancy rate of area "8" of the MNI structural atlas within the VOI. This area represents the temporal lobe.
MNI_str_loc.10	Occupancy rate of area "8" of the MNI structural atlas within the VOI. This area represents the thalamus.
HrvdOxf_loc.01	Occupancy rate of area "0" of the Harvard-Oxford cortical and structural atlas within the VOI.
HrvdOxf_loc.02	Occupancy rate of area "1" of the Harvard-Oxford cortical and structural atlas within the VOI.
HrvdOxf_loc.03	Occupancy rate of area "2" of the Harvard-Oxford cortical and structural atlas within the VOI.
HrvdOxf_loc.04	Occupancy rate of area "3" of the Harvard-Oxford cortical and structural atlas within the VOI.
HrvdOxf_loc.05	Occupancy rate of area "4" of the Harvard-Oxford cortical and structural atlas within the VOI.

HrvdOxf_loc.06	Occupancy rate of area “5” of the Harvard-Oxford cortical and structural atlas within the VOI.
HrvdOxf_loc.07	Occupancy rate of area “6” of the Harvard-Oxford cortical and structural atlas within the VOI.
HrvdOxf_loc.08	Occupancy rate of area “7” of the Harvard-Oxford cortical and structural atlas within the VOI.
HrvdOxf_loc.09	Occupancy rate of area “8” of the Harvard-Oxford cortical and structural atlas within the VOI.
HrvdOxf_loc.10	Occupancy rate of area “9” of the Harvard-Oxford cortical and structural atlas within the VOI.
HrvdOxf_loc.11	Occupancy rate of area “10” of the Harvard-Oxford cortical and structural atlas within the VOI.
HrvdOxf_loc.12	Occupancy rate of area “11” of the Harvard-Oxford cortical and structural atlas within the VOI.
HrvdOxf_loc.13	Occupancy rate of area “12” of the Harvard-Oxford cortical and structural atlas within the VOI.
HrvdOxf_loc.14	Occupancy rate of area “13” of the Harvard-Oxford cortical and structural atlas within the VOI.
HrvdOxf_loc.15	Occupancy rate of area “14” of the Harvard-Oxford cortical and structural atlas within the VOI.
HrvdOxf_loc.16	Occupancy rate of area “15” of the Harvard-Oxford cortical and structural atlas within the VOI.
HrvdOxf_loc.17	Occupancy rate of area “16” of the Harvard-Oxford cortical and structural atlas within the VOI.
HrvdOxf_loc.18	Occupancy rate of area “17” of the Harvard-Oxford cortical and structural atlas within the VOI.
HrvdOxf_loc.19	Occupancy rate of area “18” of the Harvard-Oxford cortical and structural atlas within the VOI.
HrvdOxf_loc.20	Occupancy rate of area “19” of the Harvard-Oxford cortical and structural atlas within the VOI.
HrvdOxf_loc.21	Occupancy rate of area “20” of the Harvard-Oxford cortical and structural atlas within the VOI.
HrvdOxf_loc.22	Occupancy rate of area “21” of the Harvard-Oxford cortical and structural atlas within the VOI.

HrvdOxf_loc.23	Occupancy rate of area “22” of the Harvard-Oxford cortical and structural atlas within the VOI.
HrvdOxf_loc.24	Occupancy rate of area “23” of the Harvard-Oxford cortical and structural atlas within the VOI.
HrvdOxf_loc.25	Occupancy rate of area “24” of the Harvard-Oxford cortical and structural atlas within the VOI.
HrvdOxf_loc.26	Occupancy rate of area “25” of the Harvard-Oxford cortical and structural atlas within the VOI.
HrvdOxf_loc.27	Occupancy rate of area “26” of the Harvard-Oxford cortical and structural atlas within the VOI.
HrvdOxf_loc.28	Occupancy rate of area “27” of the Harvard-Oxford cortical and structural atlas within the VOI.
HrvdOxf_loc.29	Occupancy rate of area “28” of the Harvard-Oxford cortical and structural atlas within the VOI.
HrvdOxf_loc.30	Occupancy rate of area “29” of the Harvard-Oxford cortical and structural atlas within the VOI.
HrvdOxf_loc.31	Occupancy rate of area “30” of the Harvard-Oxford cortical and structural atlas within the VOI.
HrvdOxf_loc.32	Occupancy rate of area “31” of the Harvard-Oxford cortical and structural atlas within the VOI.
HrvdOxf_loc.33	Occupancy rate of area “32” of the Harvard-Oxford cortical and structural atlas within the VOI.
HrvdOxf_loc.34	Occupancy rate of area “33” of the Harvard-Oxford cortical and structural atlas within the VOI.
HrvdOxf_loc.35	Occupancy rate of area “34” of the Harvard-Oxford cortical and structural atlas within the VOI.
HrvdOxf_loc.36	Occupancy rate of area “35” of the Harvard-Oxford cortical and structural atlas within the VOI.
HrvdOxf_loc.37	Occupancy rate of area “36” of the Harvard-Oxford cortical and structural atlas within the VOI.
HrvdOxf_loc.38	Occupancy rate of area “37” of the Harvard-Oxford cortical and structural atlas within the VOI.
HrvdOxf_loc.39	Occupancy rate of area “38” of the Harvard-Oxford cortical and structural atlas within the VOI.

HrvdOxf_loc.40	Occupancy rate of area “39” of the Harvard-Oxford cortical and structural atlas within the VOI.
HrvdOxf_loc.41	Occupancy rate of area “40” of the Harvard-Oxford cortical and structural atlas within the VOI.
HrvdOxf_loc.42	Occupancy rate of area “41” of the Harvard-Oxford cortical and structural atlas within the VOI.
HrvdOxf_loc.43	Occupancy rate of area “42” of the Harvard-Oxford cortical and structural atlas within the VOI.
HrvdOxf_loc.44	Occupancy rate of area “43” of the Harvard-Oxford cortical and structural atlas within the VOI.
HrvdOxf_loc.45	Occupancy rate of area “44” of the Harvard-Oxford cortical and structural atlas within the VOI.
HrvdOxf_loc.46	Occupancy rate of area “45” of the Harvard-Oxford cortical and structural atlas within the VOI.
HrvdOxf_loc.47	Occupancy rate of area “46” of the Harvard-Oxford cortical and structural atlas within the VOI.
HrvdOxf_loc.48	Occupancy rate of area “47” of the Harvard-Oxford cortical and structural atlas within the VOI.
HrvdOxf_loc.49	Occupancy rate of area “48” of the Harvard-Oxford cortical and structural atlas within the VOI.

1) Entropy(S) is calculated by the following equation where p_i stands for the frequency of the gray scale level i.

$$S = -\sum_{i=0}^{255} p_i \log_2 p_i$$

2) Prewitt filtering was performed by applying first order horizontal Gx and vertical Gy differentiation and by calculating the magnitude G, where Gx and Gy stands for the horizontal and vertical gradient of the image respectively and A for the original two-dimensional gray scale image

$$\mathrm{G}x = \begin{bmatrix} -1 & 0 & +1 \\ -1 & 0 & +1 \\ -1 & 0 & +1 \end{bmatrix} * \mathrm{A}, \quad \mathrm{G}y = \begin{bmatrix} -1 & -1 & -1 \\ 0 & 0 & 0 \\ +1 & +1 & +1 \end{bmatrix} * \mathrm{A}, \quad \mathrm{G} = \sqrt{\mathrm{G}x^2 + \mathrm{G}y^2}$$

3) Gdzscore image was created by visualizing the magnitude of enhancement calculated from both 256-leveled non-enhanced

and Gadolinium-enhanced T1-weighted images. 256-leveled Gadolinium-enhanced T1-weighted images was plotted as a function of 256-leveled non-enhanced T1-weighted images in whole brain. Linear regression fitting was applied to the data obtained, which can be expressed as follows;

$$(GdT1WI) = \alpha(T1WI) + \beta$$

where (*GdT1WI*) and (*T1WI*) are 256-leveled Gadolinium-enhanced and non-enhanced T1-weighted images. By solving α and β, one can now determine the linear correlation of 256-leveled Gadolinium-enhanced and non-enhanced T1-weighted images.
Next, the magnitude of deviation from the above solved linear regression line for any particular voxel (i) can be expressed as follows:

$$deviation_i = \frac{(GdT1WI)_i - \alpha(T1WI)_i - \beta}{\sqrt{\alpha^2 + 1}}$$

where $(GdT1WI)_i$ and $(T1WI)_i$ are the 256-leved values of voxel (i) in Gadolinium-enhanced and non-enhanced T1-weighted images.
Finally, the Gdzscore of each data point was defined as follows

$$Gdzscore_i = \frac{deviation_i - \mu}{\rho}$$

where μ and σ are the means and standard deviation of $deviation_i$ in the whole brain.

Table S2

R code for IDH mutation with location information
See code below

R code for IDH mutation with location information

```
library(glmnet)

##### data set prepare for training and validation#####
dataset00 = read.csv("dataset171101Train.csv")
dataset01 = read.csv("dataset171101Validation.csv")

outvar = "IDH.1"
dataset00[,"IDH.1"] = as.factor(paste0("group0",
dataset00[,"IDH.1"]))
dataset01[,"IDH.1"] = as.factor(paste0("group0",
dataset01[,"IDH.1"]))
table(dataset00[,"IDH.1"])
table(dataset01[,"IDH.1"])

modalities = c("T2", "T2E", "T1", "T1Gd", "Gdzscore", "FLAIR")
measures = c("Mean", "SD", "Max", "Min", "Median", "Mode",
"Entropy")
features = c(paste(rep(modalities, each=length(measures)),
measures, sep="_"), "Gdzscore_ara.of.Gd.")
Locations = paste0("Location.", 1:10)
others = c("totalsurfarea", "totalvol", "compactness01",
"compactness02", "sphericalDisproportion", "sphericity",
"surface2volumeratio")
Harvards = paste0("Harvard_", sprintf("%02d", 1:49))

allvars = c(outvar, features, Locations, Harvards, others)

tmpdataset2 = dataset00[,allvars]
tmpdataset2 = subset(tmpdataset2, complete.cases(tmpdataset2))
trainx <- as.matrix((tmpdataset2[,-1]))
trainy <- tmpdataset2[,1]

valdataset2 = dataset01[,allvars]
valdataset2 = subset(valdataset2, complete.cases(valdataset2))
validationx <- as.matrix((valdataset2[,-1]))
validationy <- valdataset2[,1]

paste("complete cases for training N =", nrow(tmpdataset2))
paste("complete cases for validation N =", nrow(valdataset2))


##### lasso regression #####
cvfit <- cv.glmnet(trainx, trainy, family="multinomial",
type.multinomial="grouped")
predytrain <- predict(cvfit, newx=trainx, type="class", s =
'lambda.min')
table(predytrain,trainy)
predyvalidation <- predict(cvfit, newx=validationx,
type="class", s = 'lambda.min')
table(predyvalidation,validationy)
plot(cvfit)
coef(cvfit, s=cvfit$lambda.1se)
```

R code for IDH mutation without location information

```
library(glmnet)

##### data set prepare for training and validation#####
dataset00 = read.csv("dataset171101Train.csv")
dataset01 = read.csv("dataset171101Validation.csv")
```

```
outvar = "IDH.1"
dataset00[,"IDH.1"] = as.factor(paste0("group0",
dataset00[,"IDH.1"]))
dataset01[,"IDH.1"] = as.factor(paste0("group0",
dataset01[,"IDH.1"]))
table(dataset00[,"IDH.1"])
table(dataset01[,"IDH.1"])

modalities = c("T2", "T2E", "T1", "T1Gd", "Gdzscore", "FLAIR")
measures = c("Mean", "SD", "Max", "Min", "Median", "Mode",
"Entropy")
features = c(paste(rep(modalities, each=length(measures)),
measures, sep="_"), "Gdzscore_ara.of.Gd.")
Locations = paste0("Location.", 1:10)
others = c("totalsurfarea", "totalvol", "compactness01",
"compactness02", "sphericalDisproportion", "sphericity",
"surface2volumeratio")
Harvards = paste0("Harvard_", sprintf("%02d", 1:49))

allvars = c(outvar, features, others)

tmpdataset2 = dataset00[,allvars]
tmpdataset2 = subset(tmpdataset2, complete.cases(tmpdataset2))
trainx <- as.matrix((tmpdataset2[,-1]))
trainy <- tmpdataset2[,1]

valdataset2 = dataset01[,allvars]
valdataset2 = subset(valdataset2, complete.cases(valdataset2))
validationx <- as.matrix((valdataset2[,-1]))
validationy <- valdataset2[,1]

paste("complete cases for training N =", nrow(tmpdataset2))
paste("complete cases for validation N =", nrow(valdataset2))


##### lasso regression #####
cvfit <- cv.glmnet(trainx, trainy, family="multinomial",
type.multinomial="grouped")
predytrain <- predict(cvfit, newx=trainx, type="class", s =
'lambda.min')
table(predytrain,trainy)
predyvalidation <- predict(cvfit, newx=validationx,
type="class", s = 'lambda.min')
table(predyvalidation,validationy)
plot(cvfit)
coef(cvfit, s=cvfit$lambda.min)
cvfit$lambda.min
```

R code for 3 group discrimination

```
library(glmnet)

##### data set prepare for training and validation#####
dataset00 = read.csv("dataset171101Train.csv")
dataset01 = read.csv("dataset171101Validation.csv")

outvar = "X3.Groups"
dataset00[,"X3.Groups"] = as.factor(paste0("group0",
dataset00[,"X3.Groups"]))
dataset01[,"X3.Groups"] = as.factor(paste0("group0",
dataset01[,"X3.Groups"]))
table(dataset00[,"X3.Groups"])
table(dataset01[,"X3.Groups"])
```

```
modalities = c("T2", "T2E", "T1", "T1Gd", "Gdzscore", "FLAIR")
measures = c("Mean", "SD", "Max", "Min", "Median", "Mode",
"Entropy")
features = c(paste(rep(modalities, each=length(measures)),
measures, sep="_"), "Gdzscore_ara.of.Gd.")
Locations = paste0("Location.", 1:10)
others = c("totalsurfarea", "totalvol", "compactness01",
"compactness02", "sphericalDisproportion", "sphericity",
"surface2volumeratio")
Harvards = paste0("Harvard_", sprintf("%02d", 1:49))

allvars = c(outvar, features, Locations, Harvards, others)

tmpdataset2 = dataset00[,allvars]
tmpdataset2 = subset(tmpdataset2, complete.cases(tmpdataset2))
trainx <- as.matrix((tmpdataset2[,-1]))
trainy <- tmpdataset2[,1]

valdataset2 = dataset01[,allvars]
valdataset2 = subset(valdataset2, complete.cases(valdataset2))
validationx <- as.matrix((valdataset2[,-1]))
validationy <- valdataset2[,1]

paste("complete cases for training N =", nrow(tmpdataset2))
paste("complete cases for validation N =", nrow(valdataset2))


##### lasso regression #####
cvfit <- cv.glmnet(trainx, trainy, family="multinomial",
type.multinomial="grouped")
predytrain <- predict(cvfit, newx=trainx, type="class", s =
'lambda.min')
table(predytrain,trainy)
predyvalidation <- predict(cvfit, newx=validationx,
type="class", s = 'lambda.min')
table(predyvalidation,validationy)
plot(cvfit)
coef(cvfit, s=cvfit$lambda.min)
s=cvfit$lambda.min
```

Table S3

Significant variables are in red	*n*	mean	standard deviation	minimum	maximum	missing%	*IDH*-mut only (*n* = 63) mean (sd)	*IDH*, *TERT* co-mutated (*n* = 66) mean (sd)	*IDH*-wt (*n* = 70) mean (sd)	*p*
T2_Mean	199	113.3	24.3	54.4	189.2	0	121.79 (25.91)	112.27 (21.60)	106.67 (23.36)	0.001
T2_SD	199	21.2	7.5	8.2	44.5	0	22.68 (7.30)	22.47 (7.07)	18.60 (7.38)	0.001
T2_Max	199	179.2	36.9	100	255	0	183.98 (34.45)	186.27 (38.30)	168.31 (35.71)	0.008
T2_Min	199	31.5	21	1	84	0	25.84 (21.27)	27.02 (19.23)	40.86 (19.42)	<0.001
T2_Median	199	114	25.9	46	198	0	123.76 (28.52)	112.08 (22.12)	107.07 (24.34)	0.001
T2_Mode	199	116.7	30.7	38	215	0	128.54 (33.35)	113.44 (27.10)	109.06 (28.58)	0.001
T2_Entropy	199	6.2	0.5	4.9	7.3	0	6.32 (0.47)	6.38 (0.41)	6.02 (0.53)	<0.001
T2Edge_Mean	199	41	17.5	14.7	98	0	43.53 (17.67)	41.62 (14.43)	38.02 (19.67)	0.18

T2Edge_SD	199	32.2	13	10	70.8	0	34.93 (13.39)	34.19 (13.34)	27.78 (11.38)	0.002
T2Edge_Max	199	245.1	94.9	51.6	531.2	0	267.22 (88.49)	267.41 (97.28)	204.22 (85.29)	<0.001
T2Edge_Min	199	1.6	2.5	1	25.5	0	1.24 (0.52)	1.27 (0.68)	2.18 (4.12)	0.046
T2Edge_Median	199	32.8	15.8	9.5	100.4	0	34.53 (15.65)	32.63 (12.06)	31.50 (18.90)	0.544
T2Edge_Mode	199	12.8	8.4	1	70	0	12.79 (6.63)	12.59 (7.46)	12.88 (10.52)	0.98
T2Edge_Entropy	199	5.2	0.5	3.9	6.5	0	5.38 (0.52)	5.33 (0.48)	5.03 (0.52)	<0.001
T1_Mean	191	80.6	20.4	40.9	213.3	4	77.89 (19.36)	78.85 (18.45)	84.62 (22.49)	0.125
T1_SD	191	14	5.3	5	47.7	4	15.14 (6.13)	14.40 (5.45)	12.64 (3.85)	0.021
T1_Max	191	141	43.6	70	255	4	148.69 (47.59)	140.33 (41.33)	134.70 (41.31)	0.191
T1_Min	191	16.8	18	1	97	4	12.18 (12.75)	13.48 (15.28)	24.22 (21.92)	<0.001
T1_Median	191	80.9	20.5	38	216	4	77.61 (19.42)	79.21 (18.56)	85.40 (22.64)	0.073
T1_Mode	191	80.3	21.8	17	219	4	76.67 (20.62)	78.62 (18.92)	85.04 (24.69)	0.073

T1_Entropy	191	5.6	0.4	4.2	7.5	4	5.75 (0.41)	5.68 (0.45)	5.44 (0.38)	<0.001
T1Gd_Mean	189	84	22.9	15.2	164.7	5	80.08 (23.78)	81.35 (18.06)	90.25 (25.15)	0.022
T1Gd_SD	189	16	8	3.3	44.8	5	16.17 (7.52)	14.75 (7.41)	16.89 (8.80)	0.305
T1Gd_Max	189	173.7	51.6	39	255	5	180.36 (49.93)	162.94 (46.94)	177.58 (56.36)	0.13
T1Gd_Min	189	24.5	18.1	1	108	5	21.28 (16.74)	18.95 (11.68)	32.70 (21.27)	<0.001
T1Gd_Median	189	82.9	22.3	15	159	5	79.07 (23.82)	80.71 (17.28)	88.59 (24.17)	0.034
T1Gd_Mode	189	80.3	24.2	1	156	5	75.21 (25.89)	78.50 (20.29)	86.56 (24.94)	0.023
T1Gd_Entropy	189	5.7	0.6	3.7	7.1	5	5.73 (0.58)	5.62 (0.56)	5.68 (0.67)	0.63
Gdzscore_Mean	189	0.5	0.7	-0.5	3	5	0.47 (0.67)	0.48 (0.62)	0.65 (0.74)	0.219
Gdzscore_SD	189	0.5	0.4	0.1	2.1	5	0.49 (0.33)	0.50 (0.37)	0.61 (0.40)	0.135
Gdzscore_Max	189	4.7	2.1	0.4	11	5	4.91 (1.88)	4.45 (1.97)	4.71 (2.36)	0.486
Gdzscore_Min	189	-1.2	1.4	-12.3	1.2	5	-1.33 (1.42)	-1.39 (1.79)	-0.94 (0.96)	0.162
Gdzscore_Median	189	0.4	0.7	-0.5	2.6	5	0.41 (0.67)	0.40 (0.57)	0.51 (0.71)	0.589
Gdzscore_Mode	189	0	1.2	-9.7	2.5	5	-0.04 (0.94)	-0.15 (1.49)	0.11 (0.97)	0.443

Gdzscore_Entropy	189	4	0.7	2.7	6.2	5	3.93 (0.60)	3.89 (0.66)	4.16 (0.81)	0.059
FLAIR_Mean	178	126.8	31.4	54.7	213.8	10.6	124.94 (31.23)	130.98 (30.51)	124.55 (32.52)	0.461
FLAIR_SD	178	28.3	10.5	9.8	62.4	10.6	27.75 (9.77)	31.37 (10.84)	25.94 (10.15)	0.015
FLAIR_Max	178	191.2	42.7	88	255	10.6	188.03 (42.10)	201.19 (40.87)	184.64 (44.00)	0.082
FLAIR_Min	178	10.4	19	1	118	10.6	8.07 (19.15)	7.61 (12.21)	15.33 (23.00)	0.042
FLAIR_Median	178	130.7	33.1	56	229	10.6	128.46 (32.63)	135.86 (32.50)	127.85 (33.99)	0.342
FLAIR_Mode	178	142.2	48.9	50	255	10.6	138.16 (46.11)	148.32 (48.69)	140.08 (51.73)	0.49
FLAIR_Entropy	178	6.5	0.5	5.1	7.5	10.6	6.52 (0.49)	6.69 (0.44)	6.37 (0.48)	0.001
Gdzscore_area_of_Gd	189	0	0	0	0	5	0.00 (0.01)	0.00 (0.01)	0.00 (0.01)	0.974
MNI_str_loc.01	199	0.1	0.1	0	0.6	0	0.12 (0.06)	0.11 (0.06)	0.17 (0.13)	<0.001
MNI_str_loc.02	199	0	0.1	0	0.3	0	0.03 (0.05)	0.03 (0.03)	0.04 (0.07)	0.22
MNI_str_loc.03	199	0	0.1	0	1	0	0.02 (0.12)	0.00 (0.00)	0.04 (0.17)	0.222
MNI_str_loc.04	199	0.4	0.3	0	1	0	0.42 (0.35)	0.55 (0.34)	0.25 (0.29)	<0.001
MNI_str_loc.05	199	0	0.1	0	0.5	0	0.05 (0.08)	0.04 (0.07)	0.05 (0.08)	0.811

MNI_str_loc.06	199	0	0.1	0	0.5	0	0.02 (0.07)	0.01 (0.04)	0.02 (0.06)	0.291
MNI_str_loc.07	199	0.1	0.2	0	1	0	0.11 (0.18)	0.12 (0.24)	0.12 (0.19)	0.887
MNI_str_loc.08	199	0	0.1	0	0.9	0	0.03 (0.04)	0.02 (0.04)	0.05 (0.12)	0.19
MNI_str_loc.09	199	0.2	0.3	0	1	0	0.18 (0.26)	0.11 (0.24)	0.19 (0.28)	0.161
MNI_str_loc.10	199	0	0.1	0	0.8	0	0.02 (0.03)	0.01 (0.02)	0.07 (0.17)	0.001
HrvdOxf_loc.01	199	21.4	20.2	0	100	0	18.39 (14.09)	13.99 (9.10)	31.10 (27.53)	<0.001
HrvdOxf_loc.02	199	8.6	15.5	0	87.9	0	7.26 (12.83)	13.52 (18.93)	5.30 (13.06)	0.005
HrvdOxf_loc.03	199	4.5	6.5	0	37.4	0	4.41 (5.97)	3.94 (5.97)	5.03 (7.49)	0.621
HrvdOxf_loc.04	199	7.4	13	0	63.1	0	8.81 (14.14)	10.05 (15.10)	3.59 (8.00)	0.008
HrvdOxf_loc.05	199	6.1	10.7	0	87.4	0	7.52 (14.69)	8.07 (9.95)	3.06 (5.16)	0.01
HrvdOxf_loc.06	199	1.4	2.6	0	21.2	0	1.53 (2.85)	1.88 (3.26)	0.87 (1.21)	0.068
HrvdOxf_loc.07	199	1.5	2.2	0	14.8	0	1.72 (2.57)	1.56 (1.87)	1.25 (2.00)	0.436
HrvdOxf_loc.08	199	6.4	11.2	0	70.5	0	6.38 (12.42)	6.80 (12.22)	6.01 (8.92)	0.918
HrvdOxf_loc.09	199	1.9	3.4	0	21.7	0	2.32 (3.97)	1.53 (3.06)	1.78 (3.21)	0.411
HrvdOxf_loc.10	199	0.8	1.3	0	9.1	0	0.65 (0.71)	0.75 (1.35)	0.87 (1.64)	0.635
HrvdOxf_loc.11	199	1.1	2.2	0	18.1	0	1.09 (1.89)	0.99 (2.68)	1.13 (2.05)	0.928

HrvdOxf_loc.12	199	0.8	1.4	0	9.6	0	0.79 (1.11)	0.67 (1.47)	0.80 (1.48)	0.816
HrvdOxf_loc.13	199	1.9	5.1	0	43.2	0	2.07 (4.02)	1.76 (6.24)	1.90 (4.97)	0.942
HrvdOxf_loc.14	199	0.8	1.9	0	14.7	0	1.27 (2.59)	0.48 (1.45)	0.71 (1.28)	0.048
HrvdOxf_loc.15	199	0.5	0.7	0	4.5	0	0.61 (0.84)	0.37 (0.55)	0.45 (0.60)	0.119
HrvdOxf_loc.16	199	1	2.1	0	12.9	0	1.34 (2.47)	0.64 (1.86)	0.89 (1.89)	0.16
HrvdOxf_loc.17	199	0.5	0.9	0	7.1	0	0.70 (1.22)	0.31 (0.62)	0.47 (0.90)	0.065
HrvdOxf_loc.18	199	3.1	7.6	0	65.3	0	1.93 (4.20)	3.85 (8.61)	3.53 (8.85)	0.309
HrvdOxf_loc.19	199	1.2	3.2	0	23	0	1.00 (2.42)	1.17 (3.64)	1.30 (3.38)	0.871
HrvdOxf_loc.20	199	0.9	2.8	0	28.2	0	0.48 (0.79)	1.40 (4.53)	0.78 (1.61)	0.165
HrvdOxf_loc.21	199	1	2.2	0	17.3	0	0.93 (1.55)	0.97 (2.63)	1.01 (2.35)	0.977
HrvdOxf_loc.22	199	0.9	3.1	0	31.8	0	1.13 (3.24)	0.63 (1.93)	0.93 (3.82)	0.657
HrvdOxf_loc.23	199	1.7	7.8	0	75.1	0	2.05 (8.14)	2.12 (10.67)	0.96 (2.68)	0.626
HrvdOxf_loc.24	199	0.3	0.6	0	3.9	0	0.41 (0.70)	0.16 (0.12)	0.42 (0.75)	0.019
HrvdOxf_loc.25	199	0.3	0.4	0	3.7	0	0.28 (0.34)	0.21 (0.24)	0.27 (0.46)	0.495
HrvdOxf_loc.26	199	0.7	1.7	0	14.5	0	0.56 (1.03)	1.00 (2.58)	0.48 (1.01)	0.167
HrvdOxf_loc.27	199	1.6	3.4	0	24.1	0	1.58 (2.98)	2.04 (3.90)	1.34 (3.31)	0.476
HrvdOxf_loc.28	199	1.2	3.2	0	25.4	0	1.29 (3.24)	1.49 (4.16)	0.93 (2.00)	0.594

HrvdOxf_loc.29	199	3.3	6	0	40.5	0	3.84 (7.41)	4.52 (6.22)	1.71 (3.85)	0.018
HrvdOxf_loc.30	199	3.6	7.9	0	65.1	0	3.06 (4.85)	3.89 (6.97)	3.82 (10.55)	0.805
HrvdOxf_loc.31	199	1.5	3.9	0	24.5	0	1.73 (4.18)	0.40 (1.33)	2.43 (4.82)	0.008
HrvdOxf_loc.32	199	1	2.8	0	19	0	1.53 (3.58)	0.44 (1.78)	1.11 (2.75)	0.083
HrvdOxf_loc.33	199	0.2	0.3	0	2.4	0	0.22 (0.36)	0.15 (0.20)	0.22 (0.24)	0.221
HrvdOxf_loc.34	199	1.6	3.4	0	30.8	0	1.61 (2.58)	1.52 (3.14)	1.66 (4.18)	0.97
HrvdOxf_loc.35	199	0.9	2.1	0	18.9	0	1.05 (1.69)	0.44 (0.98)	1.20 (3.04)	0.089
HrvdOxf_loc.36	199	0.5	1.4	0	10.2	0	0.58 (1.17)	0.18 (0.46)	0.86 (1.98)	0.016
HrvdOxf_loc.37	199	0.5	2.1	0	22.5	0	0.66 (1.76)	0.10 (0.19)	0.84 (3.07)	0.1
HrvdOxf_loc.38	199	0.3	0.7	0	6.5	0	0.41 (0.66)	0.22 (0.49)	0.39 (0.96)	0.264
HrvdOxf_loc.39	199	0.6	1.4	0	10.5	0	0.83 (1.23)	0.30 (0.74)	0.79 (1.90)	0.05
HrvdOxf_loc.40	199	0.3	0.6	0	6.6	0	0.37 (0.59)	0.12 (0.18)	0.30 (0.91)	0.079
HrvdOxf_loc.41	199	0.2	1.2	0	16.2	0	0.14 (0.20)	0.10 (0.13)	0.37 (1.93)	0.348
HrvdOxf_loc.42	199	0.8	1.9	0	22.6	0	1.06 (2.90)	0.85 (1.30)	0.64 (1.07)	0.45
HrvdOxf_loc.43	199	1.8	3	0	18.7	0	1.63 (2.79)	1.82 (3.02)	1.94 (3.07)	0.833
HrvdOxf_loc.44	199	0.7	1.4	0	9.7	0	0.63 (0.99)	0.60 (1.45)	0.99 (1.54)	0.178
HrvdOxf_loc.45	199	1.1	3.4	0	43.9	0	0.81 (1.07)	0.74 (1.48)	1.84 (5.49)	0.114

HrvdOxf_loc.46	199	0.7	1.6	0	14.3	0	0.62 (0.89)	0.60 (1.20)	0.96 (2.30)	0.342
HrvdOxf_loc.47	199	0.7	1.4	0	8	0	0.71 (1.40)	0.60 (1.51)	0.76 (1.45)	0.815
HrvdOxf_loc.48	199	0	0.1	0	0.9	0	0.02 (0.07)	0.00 (0.01)	0.02 (0.12)	0.2
HrvdOxf_loc.49	199	0	0	0	0.1	0	0.00 (0.01)	0.00 (0.00)	0.00 (0.00)	0.342
totalsurfarea	199	15050.6	11192.4	491	49999	0	17636.05 (12180.26)	14471.79 (10192.69)	13269.36 (10892.84)	0.07
totalvol	199	94275.2	90619.7	692	401665	0	116158.73 (97729.66)	94462.20 (90880.79)	74403.57 (79775.64)	0.029
compactness01	199	73.9	38.8	6.3	168.6	0	84.77 (38.38)	77.32 (38.09)	60.89 (36.62)	0.001
compactness02	199	0	0	0	0.1	0	0.00 (0.00)	0.00 (0.00)	0.00 (0.01)	0.017
spherical_disproportion	199	1.6	0.2	1.2	2.3	0	1.57 (0.21)	1.53 (0.13)	1.64 (0.25)	0.009
sphericity	199	2	0.2	1.3	2.5	0	2.03 (0.24)	2.07 (0.16)	1.96 (0.28)	0.031
surface_volume_ratio	199	0.2	0.1	0.1	0.7	0	0.19 (0.06)	0.19 (0.05)	0.26 (0.12)	<0.001

Fig. S1. Location mapping on MNI152 in the axial plane. Color-coded voxel-wise lesion mapping of the three different molecular subtypes of this cohort. Frequency of the locations of the brain affected by each molecular subtype are color coded as indicated.

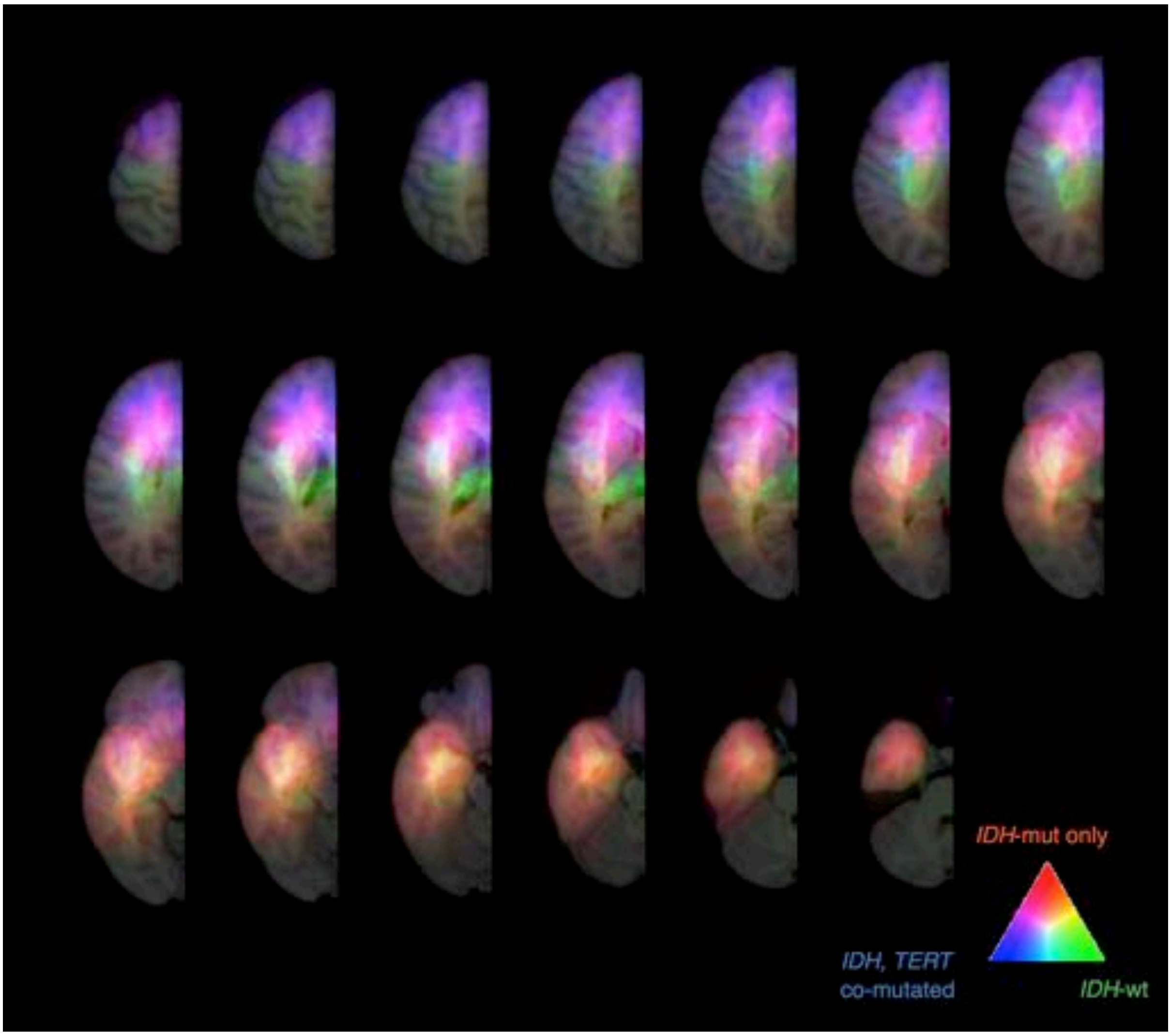

Fig. S2. Location mapping on MNI152 in the coronal plane. Color-coded voxel-wise lesion mapping of the three different molecular subtypes of this cohort. Frequency of the locations of the brain affected by each molecular subtype are color coded as indicated.

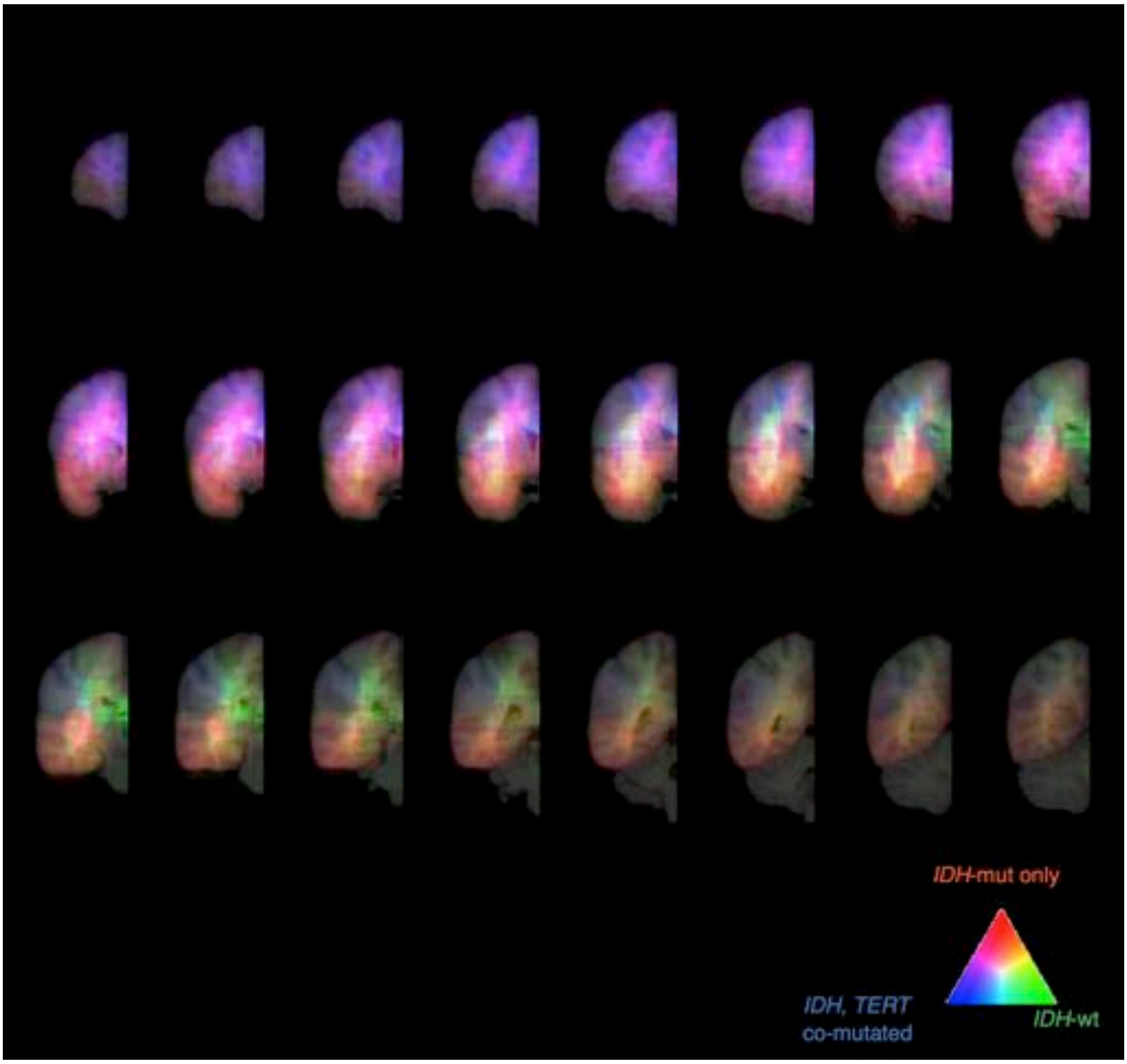

Fig. S3. Location mapping on MNI152 in the sagittal plane. Color-coded voxel-wise lesion mapping of the three different molecular subtypes of this cohort. Frequency of the locations of the brain affected by each molecular subtype are color coded as indicated.

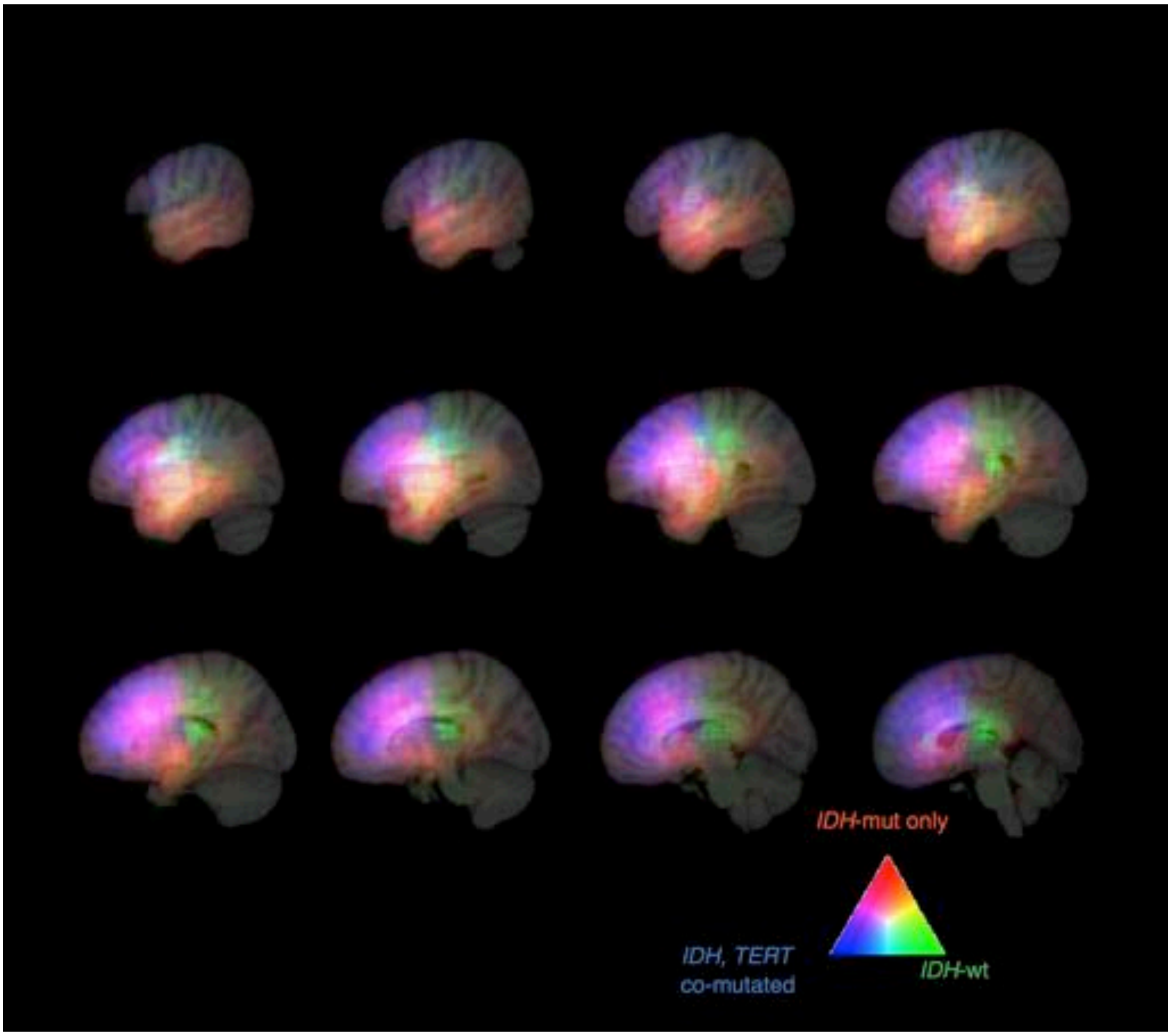

Fig. S4. Random permutation analysis mapping in the axial plane. Random permutation analysis shows locations that exhibited statistically significant differences in spatial distribution of the lesion among the three different molecular subtypes of the analyzed cohort.

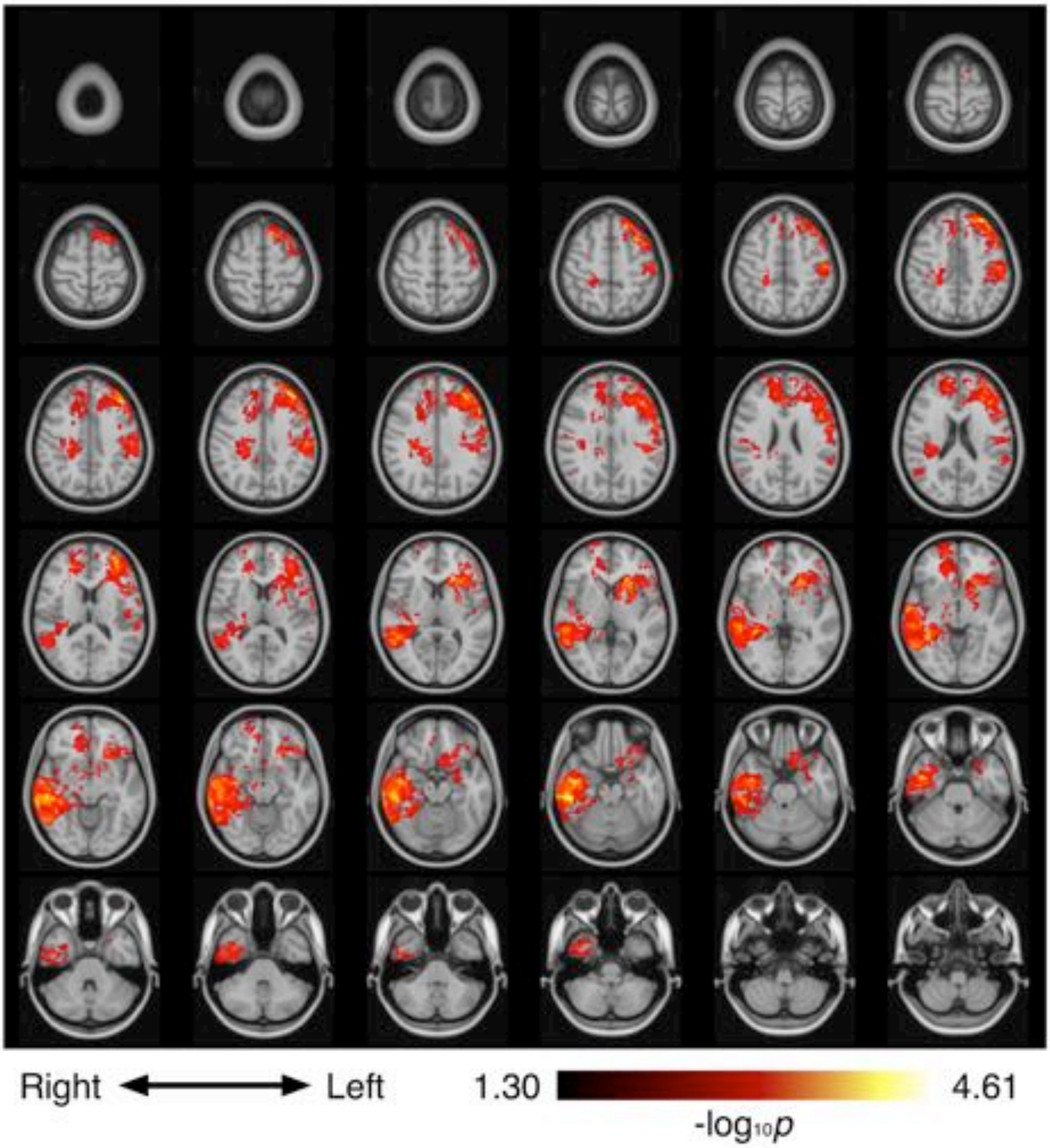

Supplement: Supplementary file 1 — Supplementary information [file 41598_2018_30273_MOESM1_ESM.pdf]
